# Asymmetric Synthesis of Tailor-Made Amino Acids Using Chiral Ni(II) Complexes of Schiff Bases. An Update of the Recent Literature

**DOI:** 10.3390/molecules25122739

**Published:** 2020-06-12

**Authors:** Yupiao Zou, Jianlin Han, Ashot S. Saghyan, Anna F. Mkrtchyan, Hiroyuki Konno, Hiroki Moriwaki, Kunisuke Izawa, Vadim A. Soloshonok

**Affiliations:** 1 Jiangsu Co-Innovation Center of Efficient Processing and Utilization of Forest Resources, College of Chemical Engineering, Nanjing Forestry University, Nanjing 210037, China; zouyupiaog1@163.com; 2 Institute of Pharmacy, Yerevan State University, 1 Alex Manoogian Str., Yerevan 0025, Armenia; saghyan@ysu.am (A.S.S.); anna_mkrtchyan@ysu.am (A.F.M.); 3 Scientific and Production Center “Armbiotechnology” of NAS RA, 14 Gyurjyan Str., Yerevan 0056, Armenia; 4 Department of Biological Engineering, Graduate School of Science and Engineering, Yamagata University, Yonezawa, Yamagata 992-8510, Japan; konno@yz.yamagata-u.ac.jp; 5 Hamari Chemical Ltd., 1-4-29 Kunijima, Higashi-Yodogawa-ku, Osaka 533-0024, Japan; hiroki-moriwaki@hamari.co.jp (H.M.); kunisuke-izawa@hamari.co.jp (K.I.); 6 Department of Organic Chemistry I, Faculty of Chemistry, University of the Basque Country UPV/EHU, Paseo Manuel Lardizábal 3, 20018 San Sebastián, Spain; 7 IKERBASQUE, Basque Foundation for Science, Alameda Urquijo 36-5, Plaza Bizkaia, 48011 Bilbao, Spain

**Keywords:** tailor-made amino acids, Schiff bases, asymmetric synthesis, chiral tridentate ligands, square-planar Ni(II) complexes

## Abstract

Tailor-made amino acids are indispensable structural components of modern medicinal chemistry and drug design. Consequently, stereo-controlled preparation of amino acids is the area of high research activity. Over last decade, application of Ni(II) complexes of Schiff bases derived from glycine and chiral tridentate ligands has emerged as a leading methodology for the synthesis of various structural types of amino acids. This review article summarizes examples of asymmetric synthesis of tailor-made α-amino acids via the corresponding Ni(II) complexes, reported in the literature over the last four years. A general overview of this methodology is provided, with the emphasis given to practicality, scalability, cost-structure and recyclability of the chiral tridentate ligands.

## 1. Introduction

**Scheme 1:**
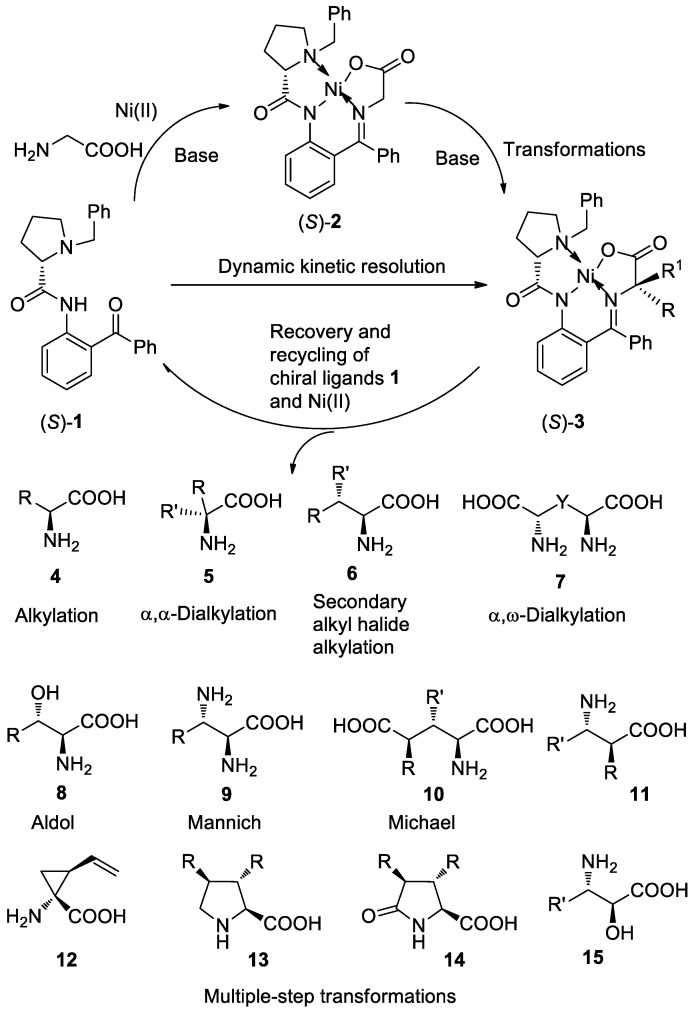
Asymmetric synthesis of tailor-made amino acid via Ni(II) complexes of Schiff bases.

Tailor-made amino acids (AAs) [1] are essential structural features of modern medicinal chemistry and drug design. The presence of orthogonal amino and carboxyl groups in adaptable proximity (α-, β-, γ-, etc.), combined with elements of chirality and diverse side chains, provides an exceptional three-dimensional structural framework with a high degree of chemical/biological functionality. These properties make AAs ideally suited for syntheses of complex molecules, highly diverse elements for SAR campaigns, basic components of modern pharmaceuticals [2,3,4,5]. In fact, over 30% of small-molecule drugs contain residues of tailor-made AAs [2,6,7,8], while peptidomimetics and peptide class drugs are fully based on AAs [9,10,11,12]. Consequently, the interest in development of synthetic approached for the preparation of tailor-made AA is at an all-time high [13,14,15,16,17,18,19,20,21,22]. Over the last decade, transformations of chiral Ni(II) complexes of Schiff bases derived from tridentate ligands and AAs (Scheme 1), have emerged as a leading methodology for asymmetric synthesis of tailor-made AAs [23,24]. 

As presented in Scheme 1, the original Belokon’ [25,26,27] tridentate ligand (*S*)-**1**, or its more recent modifications [23,24,28,29] can be efficiently used for direct reactions with unprotected AAs to obtain Ni complexes **3**. This process allows for efficient deracemization, dynamic thermodynamic resolution, or (*S*) to (*R*) interconversion of unprotected α- [30,31,32,33] and β-AAs [34]. A more general approach includes preparation of the glycine Schiff base Ni(II) complex **2**, followed by transformation of the glycine moiety. Most frequently used reaction types include, but not limited to, alkyl halide alkylations [35,36] **4**, α,α-dialkylations [37,38] **5**, secondary alkyl halide alkylations [39] **6**, α,ω-dialkylations [40,41] **7**, aldol [42,43] **8**, Mannich [44,45] **9** and Michael [46,47] **10** addition reactions. Synthesis of various β-AAs of types **11** [48] and **15** [49], as well as multiple step processes allowing access to several types of cyclic derivatives **12**–**14**, can be conveniently performed [50,51,52]. 

Application of Ni(II) complexes methodology for general asymmetric synthesis of tailor-made AAs [53,54,55], as well as special types of AAs such as fluorine-containing [56] and isotope-labelled [57], was previously reported in the corresponding reviews. The most recent update of this methodology [58], covering the literature from 2013 through the end of 2016, was published in 2017. In this review article, we summarize examples of asymmetric synthesis of tailor-made α-AAs via the corresponding Ni(II) complexes, reported in the literature over the last four years. Special emphasis is given to the aspects of practicality, scalability, cost-structure and recyclability of chiral tridentate ligands.

## 2. Large-Scale Synthesis of Chiral Ligands and Gly Schiff Base Complexes

**Figure 1:**
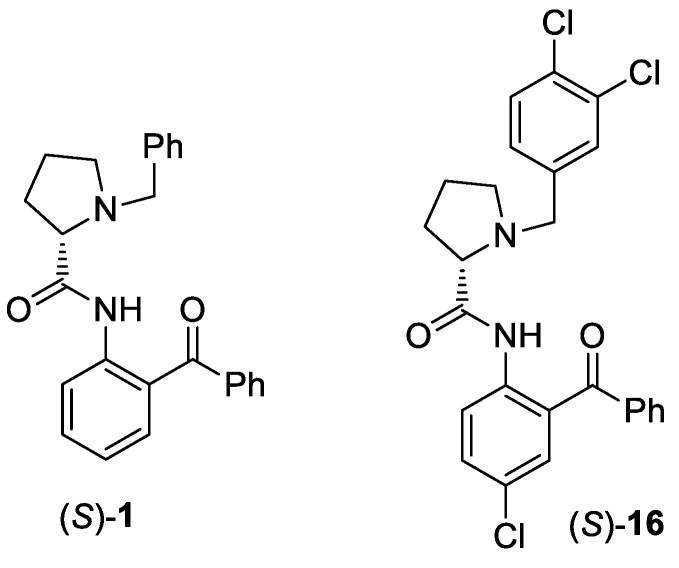
Chiral tridentate ligands.

The use of Ni(II) complexes of Gly or Ala-derived Schiff bases has been demonstrated to be one of the most efficient and practical methods for the synthesis of tailor-made AAs under simple and mild conditions. Thus, considerable efforts on the development of chiral tridentate ligands based on (*R*)- or (*S*)-*N*-(2-benzoylphenyl)-1-benzylpyrrolidine-2-carboxamide (**1**) [25,26,27] have been made to improve their stereo-controlling properties (Figure 1) [54,55,56,57].

Recently, an important achievement in this area was the discovery of the Soloshonok ligand **16** (Figure 1). This new proline-derived ligand **16**, (*R*)- and (*S)*-*N*-(2-benzoyl-4-chlorophenyl)-1-(3,4-dichlorobenzyl)pyrrolidine-2-carboxamide demonstrates excellent stereo-controlling ability and easily recyclable property, which has been successfully used in the chemical dynamic kinetic resolutions of α- and β-amino acids [32,34].

**Scheme 2:**
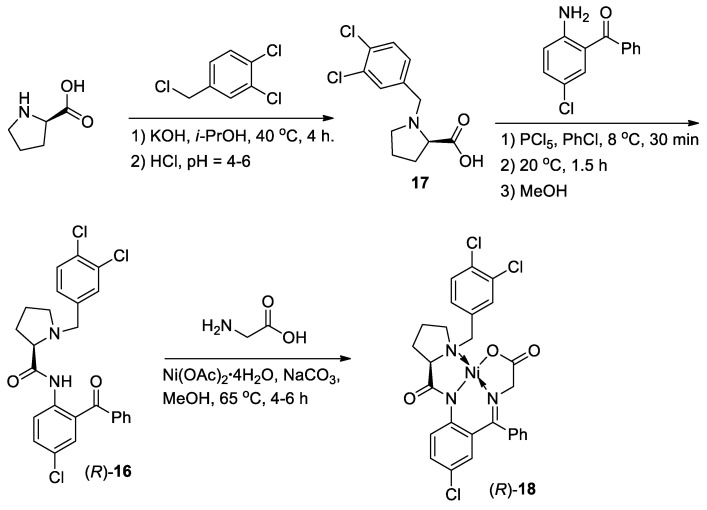
Large-scale synthesis of ligand **16** and complex **18**.

In 2017, a scale-up synthetic method for the preparation of this trichlorinated ligand **16** was reported (Scheme 2), which used commercially available D-proline as the starting material [59]. 

The reaction between D-proline and 3,4-dichlorobenzyl chloride was carried out in presence of inorganic base KOH with *i*-PrOH as a solvent at 40 °C for 4 h, followed by the treatment of hydrochloric acid to pH of 4–6, resulting in (3,4-dichlorobenzyl)-D-proline (**17**) as an off-white powder in 88% yield. The reaction of compound **17** with 2-amino-5-chlorobenzophenone with the use of PCl_5_ in chlorobenzene afforded the ligand **16** in 81% yield (total crude yield). The crude product **16** could be recrystallized from MeOH to the fine white needles. In particular, this method was reliably reproduced on a kg scale. In the initial study on the synthesis of the Ni complex **18** from the ligand **16**, an inorganic base, Na_2_CO_3_ was used. The main disadvantage of this methodology is that the reaction mixture is quite viscous for stirring at the initial stage. Furthermore, significant gas releases from the system during the workup step.

**Scheme 3:**
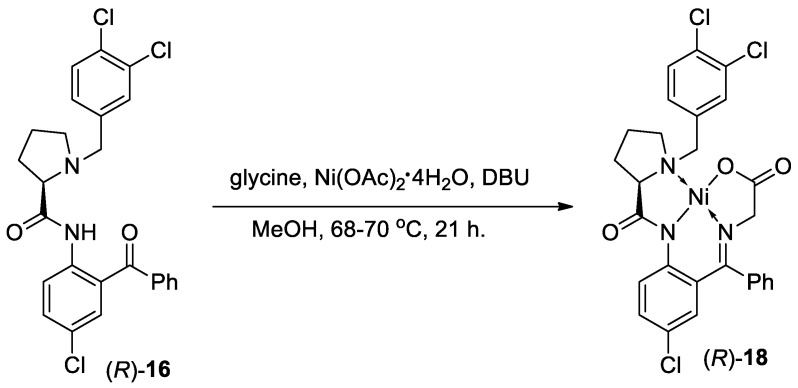
Synthesis of Ni(II) complex **18** from **16**.

In 2020, Romoff, Soloshonok, and co-workers developed an improved synthetic method for the synthesis of Ni complex **18** with the use of the soluble organic base 1,8-diazabicyclo[5.4.0]undec-7-ene (DBU) in the assembly step. Stirring (*S*)- and (*R*)-**16**, glycine, Ni(OAc)_2_. 4H_2_O and DBU in MeOH in a 50 L jacketed glass reactor at 68–70 °C for 21 h afforded the desired Ni complex **18** as an orange solid in 98.9% yield with >99% chemical purity. In this report, the chiral integrity of Ni complex **18** was also carefully examined, and the results indicate that no detectable undesirable enantiomer in the product was found under the improved reaction conditions (>99.5% chiral purity by chiral HPLC) (Scheme 3) [23]. It should be mentioned that this method could be conducted on a multi-kilogram scale with high efficiency, which demonstrates high utility of this method for the synthesis of tailor-made AAs.

**Figure 2:**
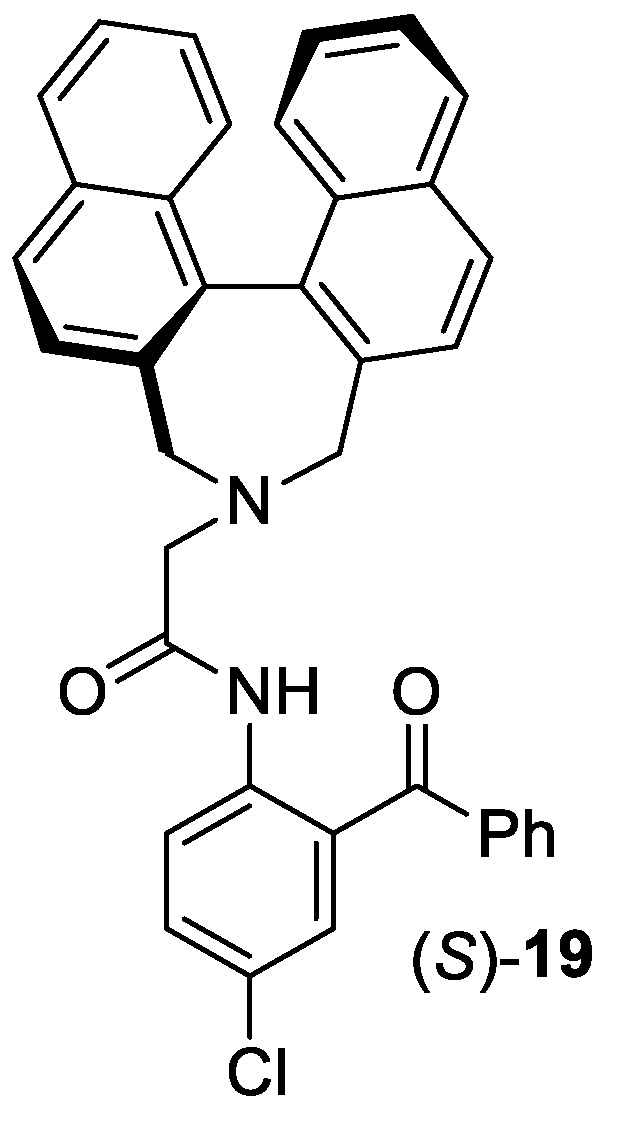
Structure of Hamari ligand.

Another chiral tridentate ligand, (3*Z*,5*Z*)-2,7-dihydro-1*H*-azepine-derived Hamari ligand **19**, also has been developed for the synthesis of AAs. Comparing with the Soloshonok ligand **16**, Hamari ligand contains a chiral binaphthalene based azepine moiety (Figure 2). 

In particular, Hamari ligand **19** is chemically stable axial chirality, and no racemization was found during the related reactions. Actually, Hamari ligand has been successfully applied in several asymmetric reactions with excellent stereoselectivities, including dynamic kinetic resolution of α-amino acids and alkylation reaction of its related Ni(II) complex [31,60].

**Scheme 4:**
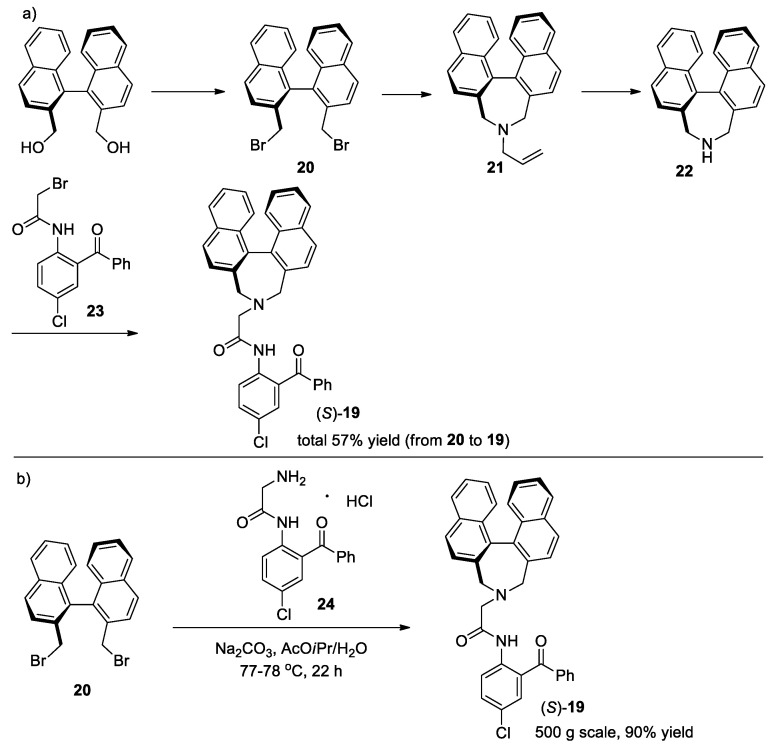
Methods for the synthesis of Hamari ligand **19** ((**a**) synthetic method from chiral binaphthol; (**b**) synthetic method via a one-step substitution alkylation reaction).

In 2014, Hamari developed a four-step synthetic method for the synthesis of ligand **19** from the commercially available chiral binaphthol (Scheme 4a) [60], which was through the sequence of bromination, alkylation with allylamine, removal of allyl protecting group and alkylation with a total 57% yield for the last three steps. This method suffered from the use of silica gel column purifications, which limited its application in large-scale manufacturing.

In 2019, an improved method for the preparation of Hamari ligand **19** was developed via a one-step substitution alkylation reaction of bis-bromide **20** by 2-amino-*N*-(2-benzoyl-4-chlorophenyl)acetamide hydrochloric salt (**24**) [61]. After careful optimization of reaction conditions, excellent yield (90%) was obtained when the reaction was conducted with the use of 6 equiv of Na_2_CO_3_, 4 volumes of AcO*i*Pr and 6 volumes of water at 78 °C for 22 h. Furthermore, this reaction could be performed in a large-scale (500 g) preparation (Scheme 4b).

**Scheme 5:**
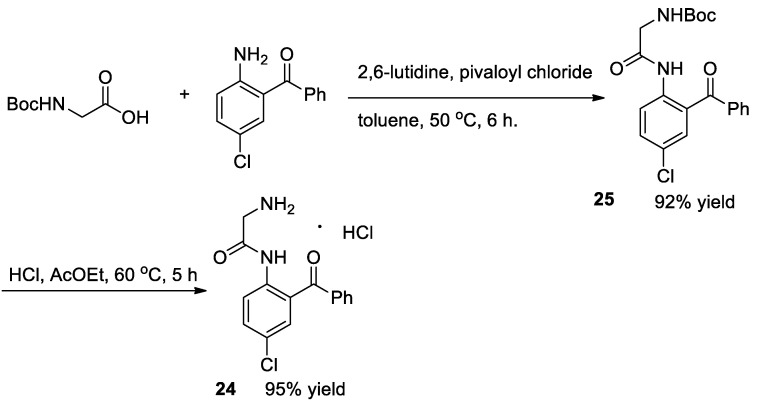
Preparation of glycinamide hydrochloride **24**.

A two-step method for the synthesis of glycinamide hydrochloride **24** was presented in Scheme 5, which used Boc-glycine as the starting material. The reaction of Boc-glycine with 2-amino-5-chlorobenzephenone in presence of pivaloyl chloride with 2,6-lutidine as a base at 50 °C for 6 h afforded the glycinamide **25** with 92% chemical yield. Boc-protecting group in glycinamide **25** was removed in the presence of HCl for 5 h at 60 °C to give the compound **24** in 95% yield.

## 3. Asymmetric Synthesis of α-Deuterated-α-Amino Acids

α-Deuterated AAs belong to a special type of isotopically labeled compounds, and also play important roles in the biochemical researches. Thus, development of mild and efficient strategy for the preparation of these compounds becomes necessary and urgent. In 2017, Takeda and co-authors successfully used the three tridentate ligands **1**, **16** and **19** in the synthesis of α-D-α-amino acids via dynamic kinetic resolution of the corresponding Ni(II) complexes [62].

**Scheme 6:**
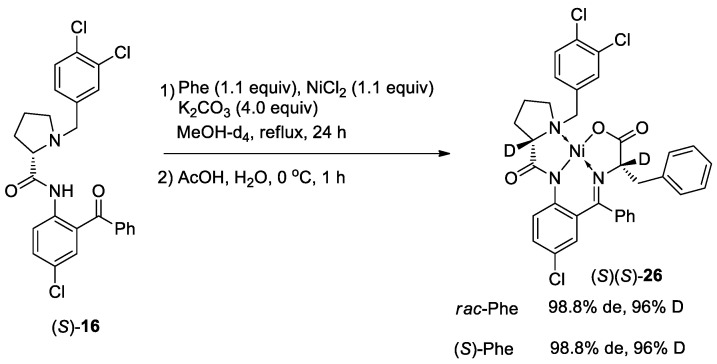
Dynamic kinetic/thermodynamic resolution of phenylalanine with concurrent deuteration using ligand (*S*)-**16**.

First, they investigated the reaction time with racemic valine as a model substrate via dynamic kinetic/thermodynamic resolution in the presence of K_2_CO_3_. Moderate degree (69%) of deuteration was observed for the ligand **19** after 24 h, while 96% deuteration degree was found for the Soloshonok ligand **16** after 20 h and 95% degree was found for the ligand **1** after 24 h. Then, the optimized ligand **16** was used for the preparation of α-deuterated Phe-derived Ni complex under the standard conditions. Both the racemic and (*S*)-Phe worked very well in the corresponding (*S*)(*S*)-**26** in excellent diastereoselectivity and high deuteration degree (98.8% d.e., 96% D). It should be mentioned that further treatment of (*S*)(*S*)-**26** by NaOMe/MeOH-*d*_4_ under reflux for 2 h could result in the nearly complete deuteration product (99% D) (Scheme 6).

**Scheme 7:**
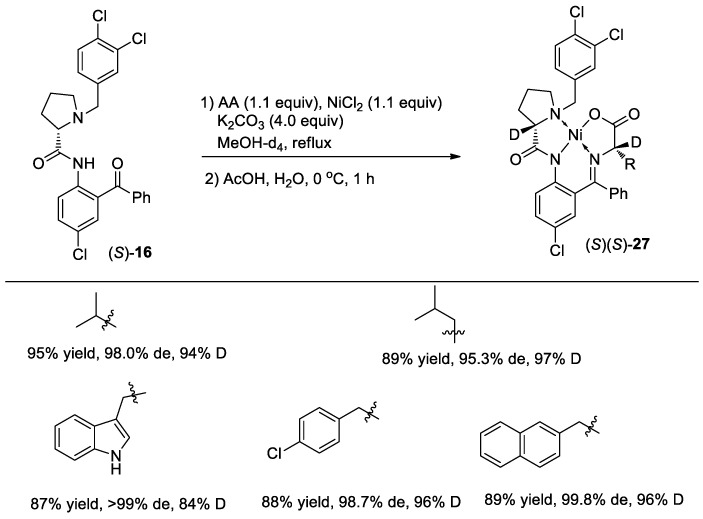
Preparation of complex (*S*)(*S*)-**27**.

Besides phenylalanine, five other AAs, including valine, leucine, tryptophan, *p*-chloro-phenylalanine and 2-(naphthyl)alanine, were tried as the substrates under the standard conditions (Scheme 7). As shown in Scheme 7, all five of these reactions gave excellent results with uniformly excellent yields (87–95%), diastereoselectivity (95–99%) and degree of deuteration (84–97%). All of these results indicate that this reaction provides an efficient and convenient way to α-deuterated AAs.

## 4. Alkyl Halide Alkylations

**Scheme 8:**
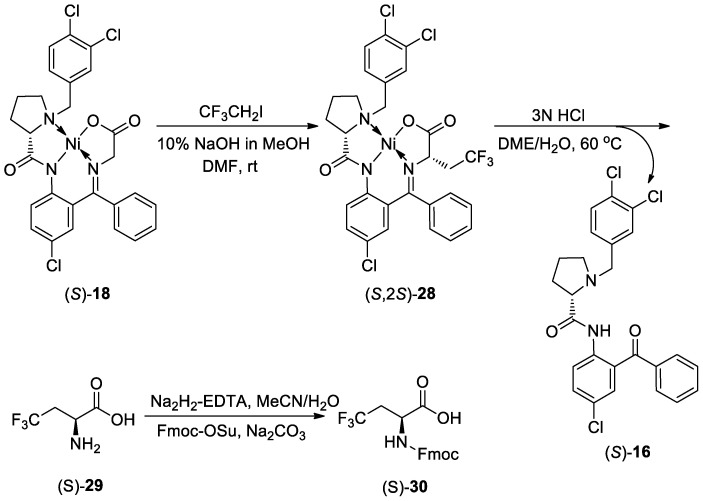
Synthesis of (*S*)-2-amino-4,4,4-trifluorobutanoic acid.

In 2019, Han and co-workers disclosed a facile and efficient protocol for the synthesis of (*S*)-2-amino-4,4,4-trifluorobutanoic acid (Scheme 8) [63]. This strategy contains three steps without any purification of the intermediates. The first step is the alkylation of the chiral Ni complex (*S*)-**18** via the nucleophilic substitution with 1.1 equivalent of trifluoroethyl iodide in DMF at room temperature. 

The intermediate (*S*,2*S*)-**28** was obtained in good yield accompanied by minor byproducts such as bis-alkylated product and decomposition of the starting material (*S*)-**18**. The disassembly of the nickel complex (*S*,2*S*)-**28** was carried out in the presence of 3N aqueous HCl at 60 °C. This transformation could easily be monitored by checking the color. Disappearance of the typical red-orange color demonstrates the accomplishment of the disassembly. The final step is the protection of the free amino group. After chelating the Ni(II) ions by ethylene diamine tetraacetic acid (EDTA) and treating the system with 9-fluorenylmethyl *N*-succinimidyl carbonate (Fmoc-OSu), *N*-Fmoc protected amino acid derivative (*S*)-**30** was formed with 74% yield, which could be easily purified by crystallization leading to 98.6% chemical purity and 97.8% ee.

Then, the workers continued their studies to use the procedure for large-scale synthesis [64]. The target product (*S*)-**30** could successfully be prepared in more than 150 g scale with good chemical yield and excellent diastereoselectivity. Notably, the stoichiometry of trifluoroethyl iodide was reduced from 10% to 5% excess ensuring less chemical cost. Nitrogen was used as atmosphere instead of air in the alkylation process to avoid oxidation byproducts. Furthermore, a two-step quenching of the alkylation mixture with water was used to isolate the generated intermediate (*S*,2*S*)-**28** without purification by column chromatography. For disassembly of the alkylation intermediate, a higher concentration of 6*N* aqueous hydrochloride was performed instead of 3N to accelerate the transformation. It should be mentioned that after disassembly, the chiral tridentate ligand (*S*)-**16** could be reclaimed and reused for synthesis of the glycine Ni(II) Schiff base (*S*)-**18** again for many times with only about 5% loss in each cycle. By employing Fmoc-OSu, the final *N*-Fmoc protected amino acid (*S*)-**30** was obtained up to hundred-gram scale in 80% yield simply by recrystallization with toluene. This newly developed methodology was proved to be sufficiently practicable and inexpensive for the large-scale synthesis of (*S*)-2-amino-4,4,4-trifluorobutanoic acid.

**Scheme 9:**
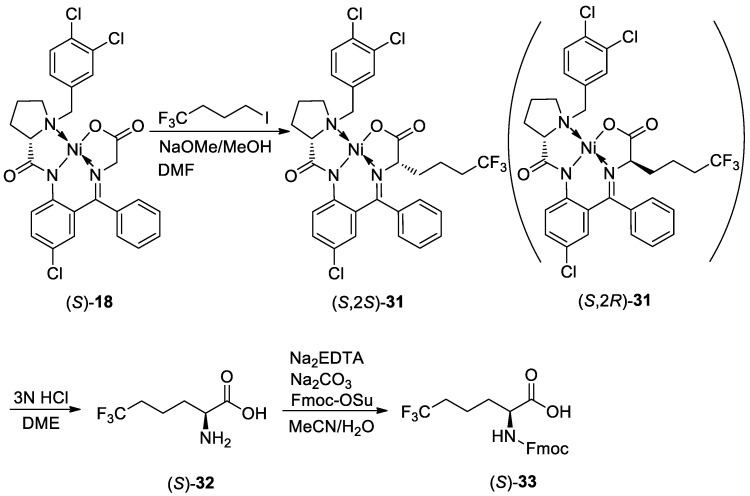
Synthesis of *N*-Fmoc-(*S*)-6,6,6-trifluoronorleucine.

Soon after, the same group reported a similar practical alkylation strategy for asymmetric synthesis of *N*-Fmoc-(*S*)-6,6,6-trifluoronorleucine (*S*)-**33** (Scheme 9) [65]. The alkylation reaction was proved to prefer homogeneous to phase-transfer catalysis conditions. When employing NaOH as base and DMSO as a solvent, the alkylation product was obtained in a quite low chemical yield (<40%) with noticeable decomposition of the starting material (*S*)-**18**. Besides, the concentration of NaOMe in MeOH affects the transformation significantly. The expected product could be obtained in a dramatically improved yield (92%) and diastereoselectivity (97:3 dr) with the use of 10% NaOMe solution in MeOH. The diastereomers (*S*,2*S*)-**31** and (*S*,2*R*)-**31** could be isolated by column chromatography. Disassembly of the major product (*S*,2*S*)-**31** proceeded smoothly under the typical conditions of 3N aqueous HCl, generating free amino acid (*S*)-**32** along with the salt of Soloshonok ligand (*S*)-**16** and Ni(II) ions. The target *N*-Fmoc-(*S*)-6,6,6-trifluoronorleucine (*S*)-**33** was formed after treatment with Fmoc-OSu and purified by recrystallization in 94% yield and 99% ee.

Fortunately, this developed route proved tolerable to provide the target *N*-Fmoc-(*S*)-6,6,6-trifluoro-norleucine more than 100 g in one batch [66]. In this protocol, the chiral ligand (*S*)-**16** as a precursor of glycine Ni(II) complex (*S*)-**18** is commercially available and could simply be reclaimed after disassembly. It was recovered quite conveniently by filtration as its hydrochloric salt. The recycled (*S*)-**16** could be reused several times with less than 10% loss in each cycle. This makes the procedure much more attractive especially for large-scale production. The major diastereomer (*S*,2*S*)-**31** could be obtained with excellent yield and diastereomeric purity after addition of water. Treating the alkylation product (*S*,2*S*)-**31** under standard disassembly and Fmoc-protection conditions, the target *N*-Fmoc-(*S*)-6,6,6-trifluoro-norleucine (*S*)-**33** was collected by recrystallization in total 83% chemical yield and >99% enantiomeric selectivity up to 105 g.

**Scheme 10:**
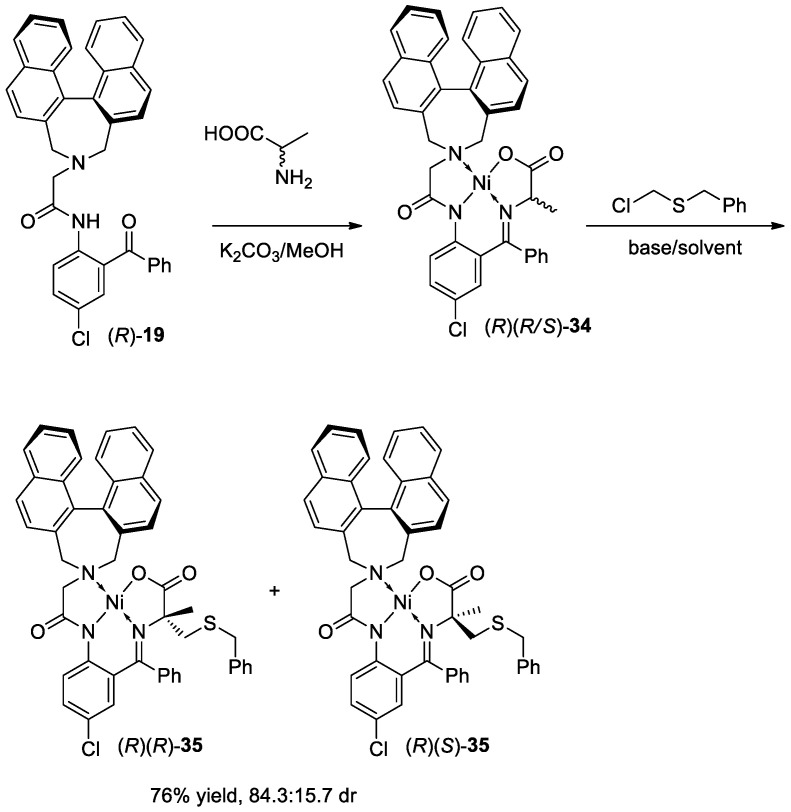
Synthesis of (*R*)(*S*)- and (*R*)(*R*)-**35** via Hamari ligand.

Moriwaki, Soloshonok and co-authors prepared (2*S*)- and (2*R*)-α-(methyl)cysteine derivatives by using the Hamari ligand and Soloshonok ligand [67]. Firstly, using chiral ligand (*R*)-**19**, the Ni complex (*R*)(*R*/*S*)-**34** was prepared in the presence of K_2_CO_3_ in MeOH. Then, benzylthiomethylation of (*R*)(*R*/*S*)-**34** with NaH as a base was successful for the preparation of the desired products (*R*)(*R*)- and (*R*)(*S*)-**35** with 76% yield and 84.3:15.7 diastereoselectivity (Scheme 10). Diastereomerically pure diastereomer (*R*)(*R*)-**35** and (*R*)(*S*)-**35** were obtained by column chromatography in 31% and 7% yield respectively.

**Scheme 11:**
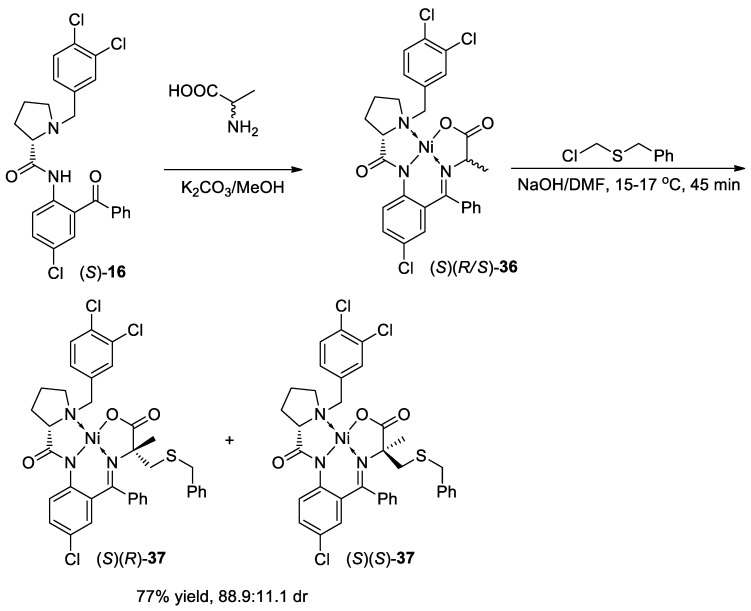
Synthesis of (*R*)(*S*)- and (*R*)(*R*)-**37** via Soloshonok ligand.

Then, using another chiral ligand (*S*)-**16** with NaOH as a base and DMF as a solvent for benzylthiomethylation successfully afforded the desired products (*S*)(*R*)- and (*S*)(*S*)-**37** in 77% isolated yield and 88.9:11.1 dr (Scheme 11). It should be mentioned that improved yield and diastereoselectivity were obtained with the use of Soloshonok ligand (*S*)-**16** for the benzylthiomethylation step.

**Scheme 12:**
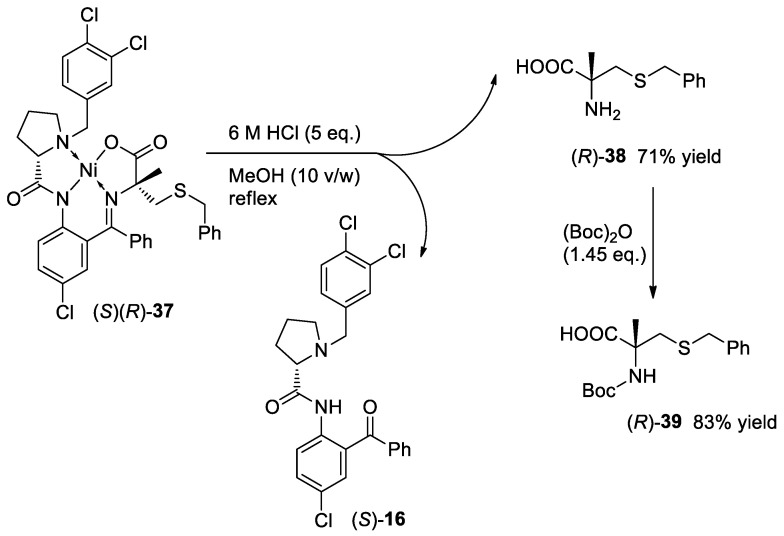
Synthesis of prepared the chiral amino acid (*R*)-**39**.

Finally, the chiral amino acid (*R*)-**39** was obtained in a total 38% yield via the disassembly of the alkylation product (*S*)(*R*)-**37** follow by Boc-protection (Scheme 12). The chiral ligand (*S*)-**16** was also easily recovered in 85% yield for the next cycle.

**Scheme 13:**
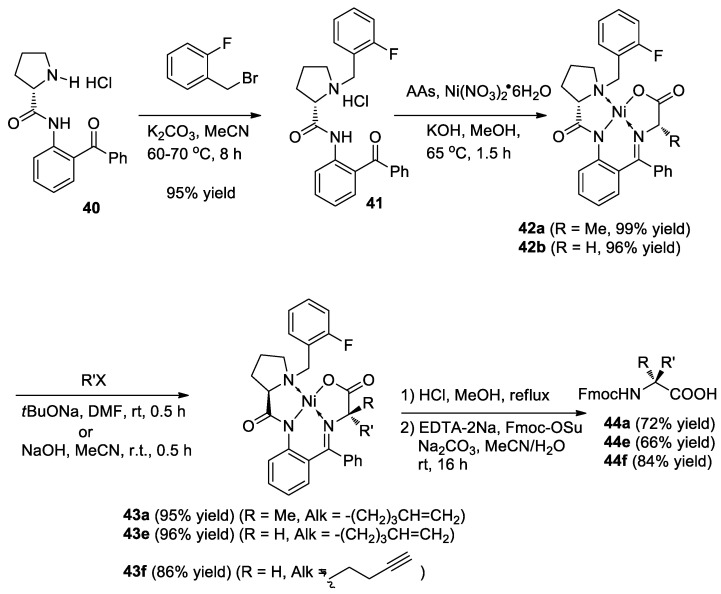
Synthesis of chiral α-substituted amino acids.

In 2017, Li and co-authors developed an improved method for the synthesis of the fluorinated chiral ligand **41** from L-proline with a total 90.6% yield and >99% enantioselectivity. Then, they used ligand **41** for the preparation of α-dialkenyl and α-alkenyl amino acids, which was shown in Scheme 13 [68]. The reaction of ligand **41** with Ni(NO_3_)_2_·6H_2_O in the presence of KOH and MeOH at 65 °C for 1.5 h afforded the Ni(II) complexes **42a** and **42b**. The Ni(II) complexes **43a** was obtained in 95% yield via the reaction of iodoalalkene with Ni(II) complexes **42a** using *t*-BuONa as a base and DMF as a solvent at room temperature for 0.5 h. The Ni(II) complexes **43e** and **43f** were obtained with 96% and 86% yield under the similar conditions with NaOH as a base and acetonitrile as a solvent. The desired amino acids **44** were obtained by the disassemble reaction of Ni(II) complexes **43a**, **43e** and **43f** in the presence of HCl. Then *N*-Fmoc protected amino acids **44** were achieved in 72%, 66% and 84% yield respectively and >99% e.e. via treatment by Fmoc-OSu and Na_2_H_2_·EDTA in MeCN/H_2_O for 16 h.

**Scheme 14:**
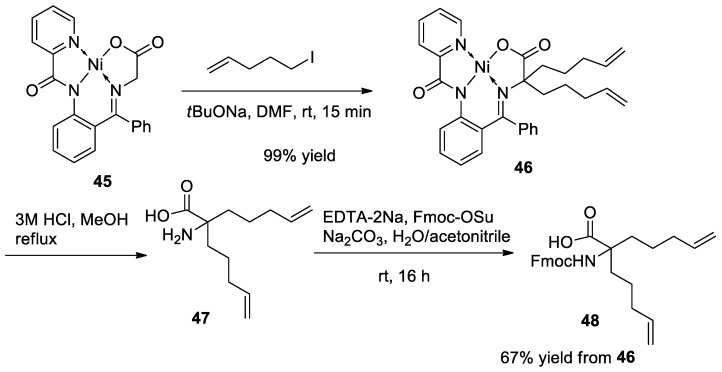
Synthesis of α,α-disubstituted amino acid.

Besides the synthesis of chiral α-substituted amino acids, the same group also used this alkylation reaction for the synthesis of achiral disubstituted amino acid **47** (Scheme 14). The alkylation of Ni complex **45** with 5-iodo-1-pentene in the presence of base at room temperature provided the Ni complex **46** in 99% yield. Then, the Fmoc-α,α-disubstituted amino acid **48** was obtained in overall 67% yield via the similar disassembly and protection processes.

## 5. Preparation via Second-Order Asymmetric Transformation Control

The second order asymmetric transformation (SOAT), which is based on labile and interconvertible stereogenic centers, represents another approach for the general synthesis of α-AAs. Compared with the regular asymmetric methodology, SOAT is not a common strategy. However, it can be used as an efficient method for the synthesis of AAs concerning operational convenience and scalability.

**Scheme 15:**
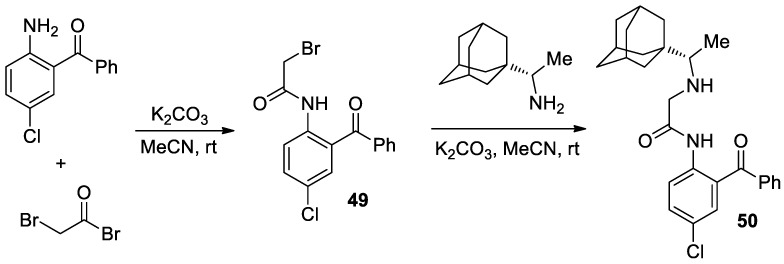
Synthesis of chiral ligand **50** containing rimantadine group.

In 2018, Takeda and co-workers [29] reported an interesting method for the asymmetric synthesis of α-amino acids via SOAT approach with the use of highly lipophilic rimantadine [1-(1-adamantyl)ethanamine] derive chiral N-H containing ligand **50**. The preparation of chiral ligand **50** was presented in Scheme 15, which was via two substitution reactions in the presence of K_2_CO_3_ at room temperature with *o*-amino-*m*-chlorobenzophenone as the starting material.

**Scheme 16:**
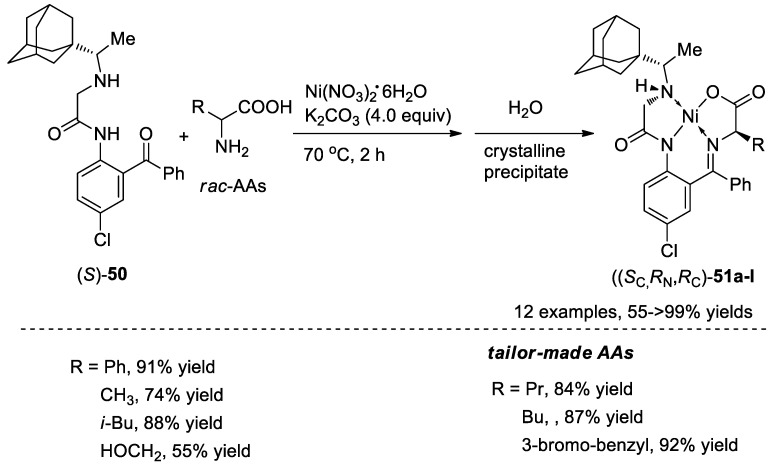
SOAT approach for the preparation of Ni complexes.

Then, this rimantadine-based chiral tridentate ligand **50** was successfully used in the dynamic kinetic resolution of unprotected racemic AAs under the SOAT conditions. In the presence of K_2_CO_3_, (*S*)-**50**, AAs and Ni(NO_3_)_2_·6H_2_O were stirred in MeOH at 70 °C for 2 h. As two new chiral centers generated in this reaction, four diastereomers may be obtained in the reaction mixture. However, diastereomerically pure products, (*S*_C_,*R*_N_,*R*_C_)-**51**, were observed via gradual formation of crystalline precipitate. The possible reason is that other diastereomers could be converted into (*S*_C_,*R*_N_,*R*_C_)-**51** in the presence of base via epimerization at the carbon center and inversion at the nitrogen center. It should be mentioned that this reaction has a wide substrate generality. Varieties of racemic natural AAs and tailor-made AAs, as well as the functional group-containing AAs, participated in this reaction very well, resulting in the corresponding Ni complexes **51** in 55–99% yields (Scheme 16).

**Scheme 17:**
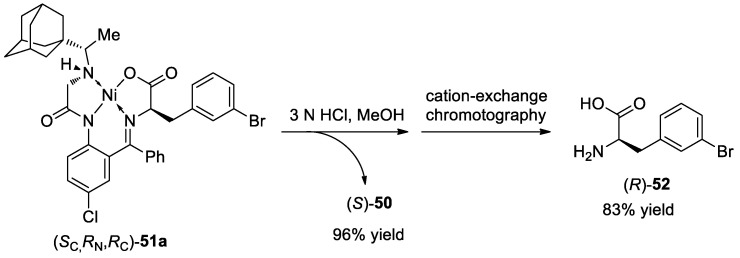
Disassembly of **51a**.

The disassembly of (*S*_C_,*R*_N_,*R*_C_)-**51a** was conducted under acidic conditions, which afforded the chiral amino acid hydrochloric salt along with the generation of chiral ligand **50** for the next cycle. Then, the amino acid (*R*)-**52** was obtained via cation-exchange chromatography in 83% yield (Scheme 17).

**Scheme 18:**
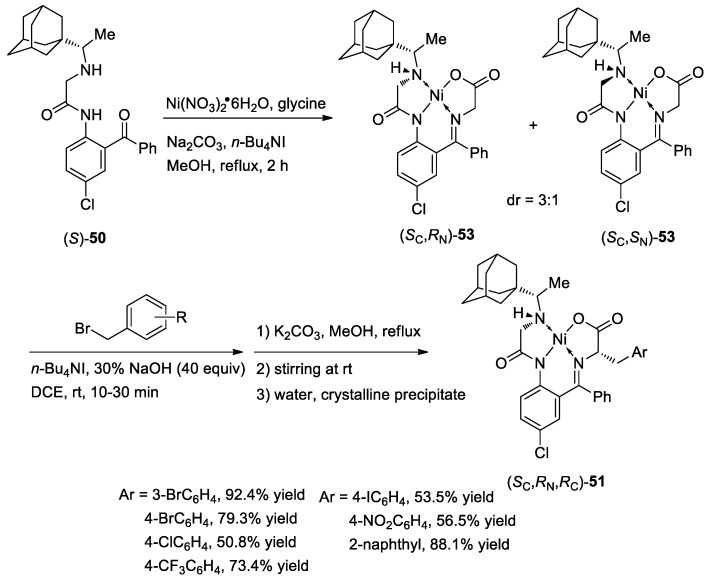
Synthesis of Ni complexes with glycine derivatives by SOAT.

Then, in 2018, the same group further extended this SOAT approach to the synthesis of phenylalanine-type tailor-made α-AA (Scheme 18) [69]. First, the Ni complex was prepared via the reaction of ligand **50** with Ni(NO_3_)_2_·6H_2_O and glycine in the presence of Na_2_CO_3_ and phase-transfer catalyst (PTC), *n*-Bu_4_NI. Two diastereomers **53** with the ratio of 3:1 were directly subjected to the alkylation reaction with benzyl bromide with the use of 40 equiv of 30% NaOH. Four diastereomers, (*S*_C_,*R*_N_,*R*_C_)-**51**, (*S*_C_,*S*_N_,*R*_C_)-**51**, (*S*_C_,*R*_N_,*S*_C_)-**51** and (*S*_C_,*S*_N_,*S*_C_)-**51** formed in this step. However, the diastereomerically pure products, (*S*_C_,*R*_N_,*R*_C_)-**51**, was obtained after refluxing in the presence of K_2_CO_3_ in MeOH for 1 h, stirring at room temperature and crystalline precipitate with the addition of water. Notably, several benzyl bromides containing different substituents, such as trifluoromethyl, bromo, chloro and nitro group were all well tolerated in this reaction in 50–92% yields.

**Scheme 19:**
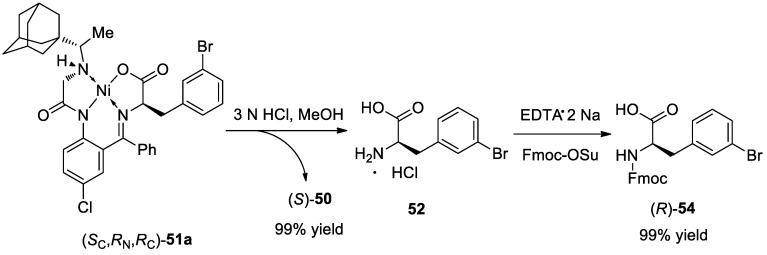
The disassembly procedure of **51a.**

The disassembly procedure for (*S*_C_,*R*_N_,*R*_C_)-**51a** was presented in Scheme 19, which was conducted under similar acidic conditions. After removal of the chiral ligand and treatment of Fmoc-Osu, the Fmoc-amino acid (*R*)-**54** were achieved in 99.1% yield and 97.1% e.e.

**Scheme 20:**
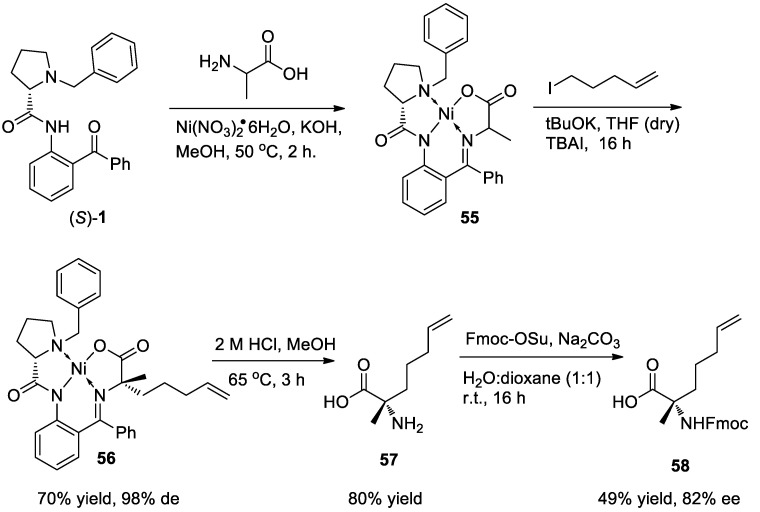
Synthesis of α,α-disubstituted amino acid derivative.

In 2019, Jamieson and co-workers disclosed a new method for the synthesis of α,α-disubstituted amino acid derivatives by using chiral tridentate ligand **1** (Scheme 20) [70]. The reaction of alanine with chiral ligand **1** afforded the Ni complex **55** in the presence of powder KOH with MeOH as a solvent at 50 °C for 2 h. The alkylation reaction of Ni complex **55** with 5-iodopentene in the presence of *t*BuOK, *n*-Bu_4_NI as PTC for 16 h afforded the desired product **56** with 70% yield and 98% ee. Product **56** was easy to disassemble in the presence of HCl at 65 °C for 3 h. Then, *N*-Fmoc-protected amino acid **58** was achieved with 49% yield and 82% e.e. by using Fmoc-Osu, Na_2_CO_3_ in dioxane/H_2_O for 16 h. Notably, although good diastereoselectivity was obtained, the decreased enantioselectivity was found in the formation of Fmoc-AAs **58**, which is mainly due to the epimerization of the α-center of the chiral auxiliary.

**Scheme 21:**
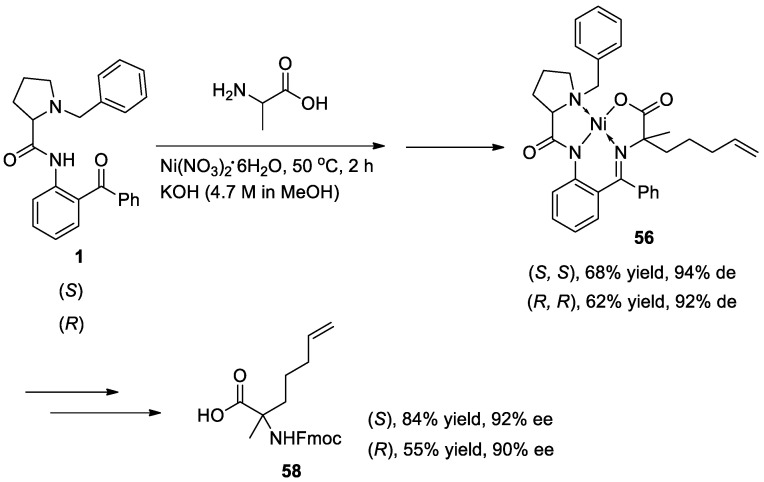
An improved synthesis route to α,α-disubstituted amino acid derivatives.

The authors then optimized their conditions for the step of the formation of complex **55**, which mainly focused on the base and reaction time. They used a KOH solution in MeOH instead of powder KOH, as well as shorter reaction time for the reaction of ligand **1** and alanine (Scheme 21). An increased enantioselectivity, as well as a good chemical yield, was obtained when (*S*)-alanine was used as the starting material (92% e.e. vs 82% e.e.).

**Scheme 22:**
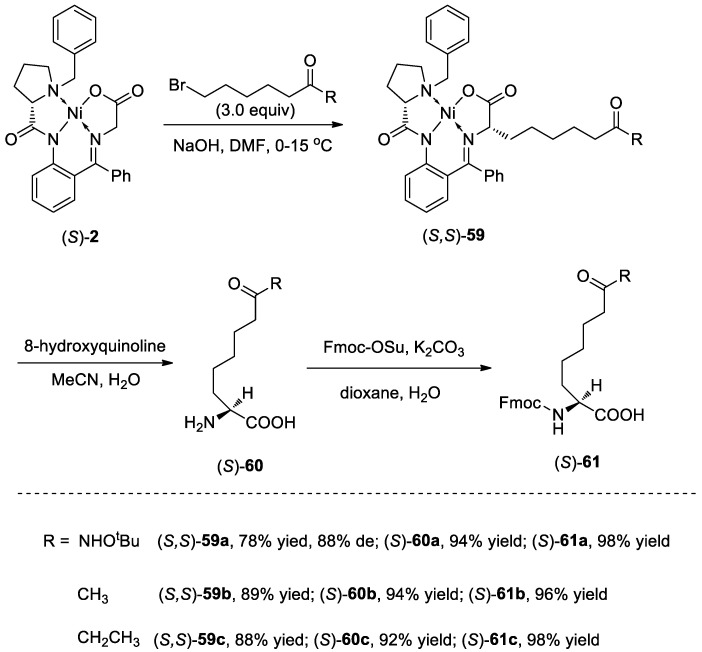
Synthesis of Fmoc amino acid **61**.

In 2019, Jamieson and co-workers successfully used the Ni complex (*S*)-**2** for the preparation of three types of amino acids, which were used in the solid-phase peptide synthesis (SPPS) of histone deacetylases (HDACs) substrate peptidomimetic inhibitors (Scheme 22) [71]. 

The initial optimization of reaction conditions indicated that the use of 3.0 equiv of alkyl bromide in the presence of sodium hydroxide in DMF at 0–15 °C provided the best reaction outcomes. The reaction of Ni complex (*S*)-**2** with 6-bromo-*N*-(*tert*-butoxy)hexanamide afforded the alkylation of product **59a** in 78% yield and 88% d.e. The disassembly of product **59a** afforded the desired amino acid **60a** in 94% yield via the treatment by 8-hydroxyquinoline in MeCN/H_2_O, which was then converted into Fmoc-L-Asu(NHO*t*Bu)-OH **61a** by reacting with Fmoc-OSu in the presence of K_2_CO_3_ and dioxane/H_2_O. Synthesis of amino acids Fmoc-L-Aon-OH **61b** and Fmoc-L-Aod-OH **61c** was achieved in excellent overall yields and high diastereoselectivities (82–84% d.e.) by using the same route with electrophiles 7-bromoheptan-2-one and 8-bromooctan-3-one as the starting materials.

It should be mentioned that the obtained Fmoc-AAs **61** were used to synthesize histone tail derived substrate peptidomimetic inhibitor (SPIs) using SPPS strategy, which provided a rapid preparation of the corresponding peptide.

**Figure 3:**
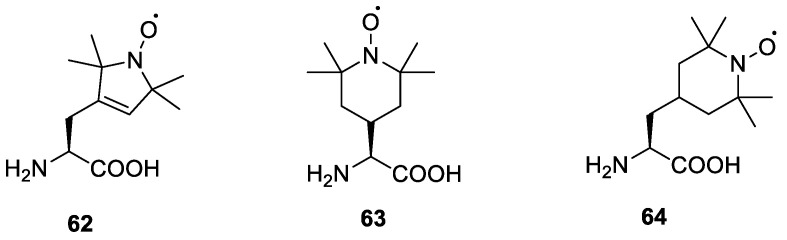
Structure of three spin-labeled target amino acids (**62–64**).

In 2019, enantioselective synthesis of three spin-labeled amino acids (**62**, **63**, **64**) (Figure 3) via the Ni complex based strategy was developed by Vederas and co-authors [72].

**Scheme 23:**
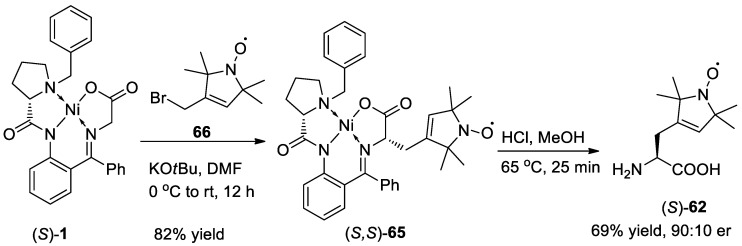
Synthesis of the spin-labeled amino acid **62**.

The synthesis of spin-labeled amino acids **62** started from the alkylation reaction between Belokon complex **1** and 2,2,6,6-tetramethyl-4-piperidinone derived bromide **66**. The Ni complex **65** was obtained in 82% yield, which was converted into the desired amino acid **62** under acidic conditions with 69% yield and 80% e.e. (Scheme 23).

**Scheme 24:**
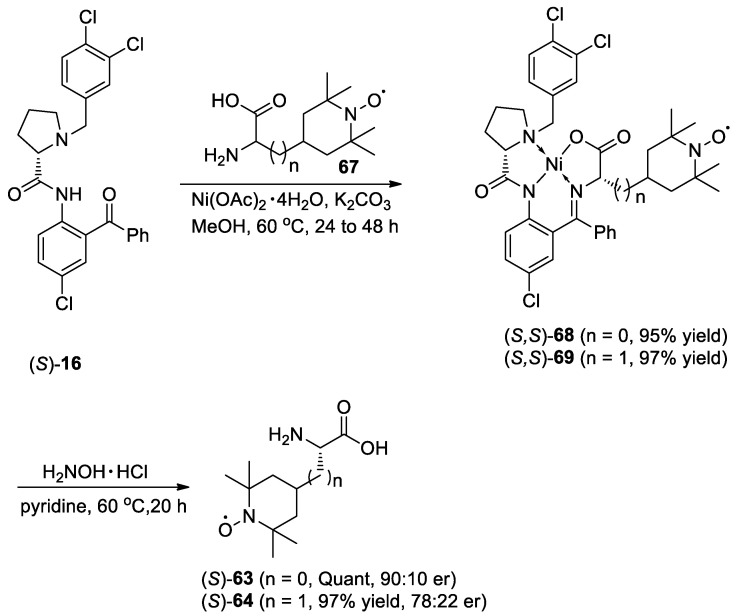
Synthesis of chiral spin-labeled amino acids **63** and **64**.

The authors also tried to apply this alkylation strategy in the preparation of the spin-labeled amino acids **63** and **64**. The results disclosed that the direct alkylation onto the Belokon complex was not successful. Thus, in this report, they used a different method, dynamic kinetic resolution (DKR) for the synthesis of spin-labeled amino acids **63** and **64** (Scheme 24). Then, they used racemic amino acids **67** to react with chiral ligand **16** and Ni(OAc)_2_ affording Ni complexes (*S*,*S*)-**68** or **69** in the presence of K_2_CO_3_ in MeOH at 60 °C for 24 to 48 h with 95% and 97% yield respectively. Finally, the chiral spin-labeled amino acids **63** and **64** were obtained with quantitative, 97% yield and 90:10, 78:22 e.e., respectively after the disassembly of Ni complex **68** or **69** in the presence of H_2_N-OH·HCl and pyridine at 60 °C for 20 h.

## 6. Addition Reactions

Asymmetric 1,6-conjugate addition reaction of Ni(II) complex of glycine with *para*-quinone methides is the simplest method to achieve the enantiomerically pure tailor-made β,β-diaryl substituted glycines [73].

**Scheme 25:**
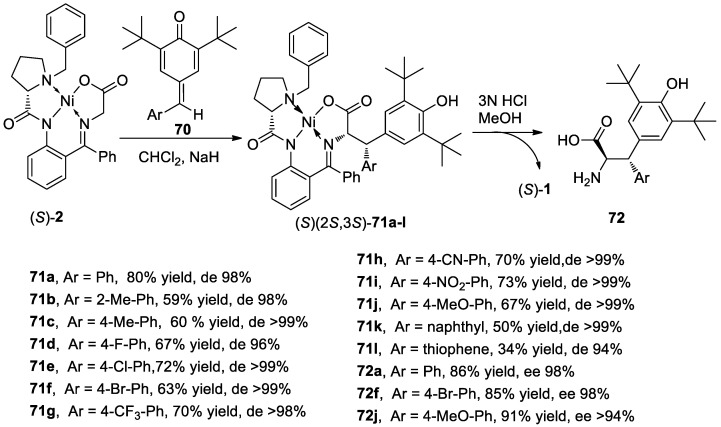
Asymmetric 1,6-conjugate addition reaction of Ni(II) complex **2**.

Recently, a convenient method for the asymmetric synthesis of tailor-made β,β-diaryl substituted glycine derivatives was developed (Scheme 25). Initially, they used the chiral Ni(II) complex **2** of glycine and ketone **70a** as model substrates to optimize the asymmetric 1,6-conjugate reaction conditions. The absolute configuration of the obtained major diastereomer complex **71** was determined as (*2S,3S*) by X-ray structural analysis. The highest chemical yield and good diastereoselectivity were observed when sodium hydride (NaH) was served as the base in CH_2_Cl_2_ (chemical yield 80%, d.e. = 98%). Then, asymmetric 1,6-conjugate addition reaction of Ni(II) complex to different *para*-quinone methides was investigated. Both electron-donating and electron-withdrawing substituents, such as methyl, methoxy, halogen, and nitro groups, were well tolerated in this asymmetric addition reaction affording the corresponding products in moderate to good yields (59–80%) and excellent diastereoselectivities (up to 99%).

**Scheme 26:**
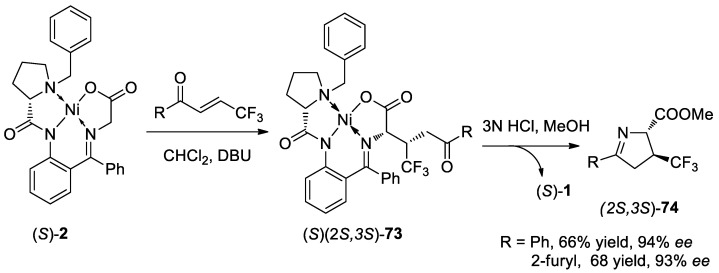
Asymmetric Michael addition of Ni(II) complex **2** with β-trifluoromethylated-α,β-unsaturated ketones.

Liu and co-authors [74] reported an asymmetric Michael addition of Ni(II) complex **2** with β-trifluoromethylated-*α*,β-unsaturated ketones for the synthesis of chiral trifluoromethyl containing heterocyclic amino acids. In this asymmetric Michael addition, major diastereomer (*S*)(2*S*,3*S*)**-73** was obtained along with other three diastereomers. After careful optimization of reaction conditions, it was found that the use of DBU in CH_2_Cl_2_ at room temperature provided the highest yield (78%) and diastereoselectivity (dr = 15/85/0/0). Then, various β-trifluoromethylated-α,β-unsaturated were screened (Scheme 26). β-Trifluoromethylated-α,β-unsaturated ketones with aryl group effectively participated in the reaction well achieving moderate chemical yields (54–78%). Moreover, fused ring and heteroaromatic ring substrates were also applicable, giving the expected products **73** with good results (68–78% yields). Remarkably, the presence of the furyl ring enhanced both the reactivity and diastereoselectivity (0/96/4/0 d.r. and 78% yield).

**Scheme 27:**
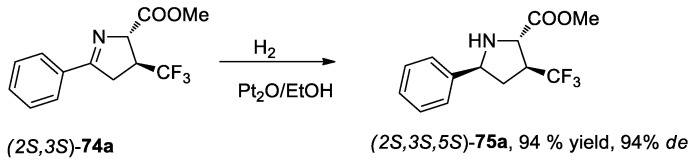
Reduction of (2*S*,3*S*)-**74a**.

Decomposition of diastereomerically pure *(S*)(2*S,*3*S*)*-***73** was conducted under the standard conditions by heating a suspension of complexes in MeOH, using 1 mol/L hydrochloric acid, which afforded the target amino acids (2*S,*3*S*)-**74** in high enantioselectivities (Scheme 26). The amino acid (2*S,*3*S*)-**74a** was further converted into trifluoromethylated proline derivative (2*S,*3*S*,5*S*)-**75a** via Pt_2_O-promoted hydrogenation (Scheme 27).

**Scheme 28:**
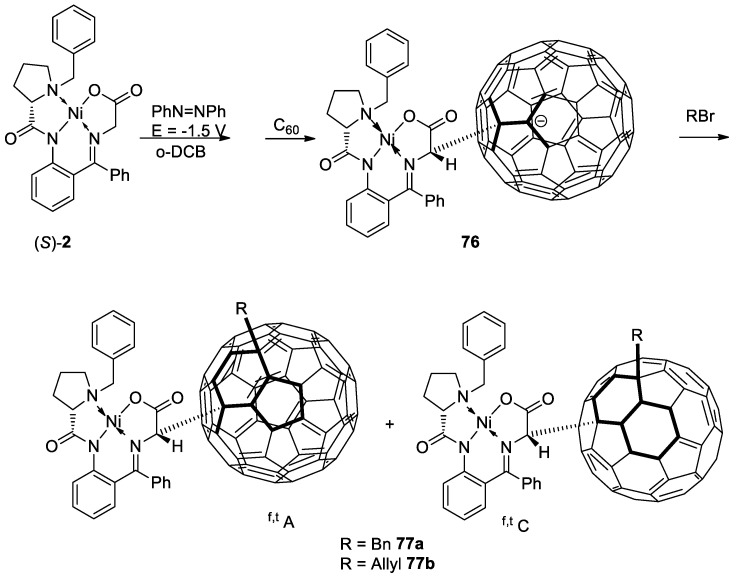
Synthesis of complexes **77a** and **77b**.

Magdesieva and co-authors [75] reported the first example of regio- and stereoselective electrosynthesis of the protected primary fulleroamino acid with double chirality (Scheme 28). Compounds (**77a** and **77b**) have three chiral centers in the addend (including the stereogenic α-amino-acid carbon center directly bound to the fullerene) and the fullerene cage itself exhibits a non-inherently chiral 1,4-functionalization pattern. Diastereo- and enantiomerically pure (^f,t^A)- and (^f,t^C)-1,4-fulleroamino acid derivatives were isolated and fully characterized. Electrochemical deprotonation of (*S*)-**2** was performed with azobenzene radical-anions (at the potential of their formation, −1.32 V vs. Ag/AgCl/KCl). After complete deprotonation of (*S*)-**2**, C_60_ was added to generate the carbanionic fullerene intermediate **76**, which underwent consecutive S_N_2 reaction with allyl or benzyl bromides.

It was found that deprotonated (*S*)-**2** and the alkyl group are attached to the fullerene cage in both isomers. The isomer ratio was 1.5:1 for the allyl and 2:1 for the benzyl auxiliary addend, and the chemical yields were 37% and 40%, respectively. Preferable formation of the 1,4-adduct was seen from the DFT calculated HOMO of the anionic intermediate formed after the nucleophilic addition of deprotonated (*S*)-**2** to C_60_. Based on the calculation and experimental CD spectra of the 1,4-regioisomers, the authors concluded that the major stereoisomer has the (*S,S*,^f,t^C) absolute configuration whereas its minor counterpart has the (*S*,*S*,^f,t^A) configuration. A voltammetric study of the (S,S,^f,t^C)-**77a** stereoisomer showed a superposition of (*S*)-**2** and C_60_ redox activity [75].

**Scheme 29:**
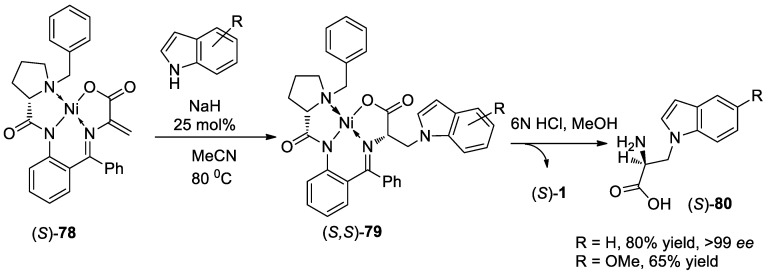
Asymmetric *aza*-Michael addition of indoles.

Larionov et al. [76] reported a selective *N-*functionalization of indoles via Michael addition of indole to chiral Ni(II) Schiff base complex **78** (Scheme 29). Ni(II) complex of the Schiff base of dehydroalanine (*S*)-**78** and indole were chosen as the model substrates to optimize reaction conditions. Good chemical yield (80%) was observed when NaH was used as the base. It was shown that indoles with electron-withdrawing substituents furnished the products **79** in moderate to good yields (55–81%). Indoles with electron-donating groups furnished the products **80** in good yields of 80% and 82% (Scheme 29).

DFT calculations helped to rationalize the observed *N*1-alkylation of the indoles over the non-observed C3-alkylation. DFT calculations results show that the top-side attack by nitrogen atom is the most favorable due to the absence of steric clashes with the benzyl group in the proline fragment. The authors concluded that the top-side attack by nitrogen atom is the most favorable leading to the desired product.

**Scheme 30:**
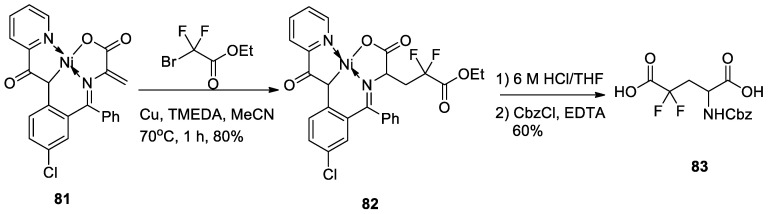
Michael-type addition reaction between achiral complex of dehydroalanine and ethyl bromodifluoroacetate.

Fluorine-containing amino acids are of growing importance in drug discovery and peptide chemistry [5,77,78]. Although a variety of synthetic methods of γ-fluorinated analogs of glutamic acid and glutamine were reported [79,80,81], there were many problematic issues such as multi-step reaction sequences and synthetically unattractive stereochemical outcome. Therefore, Michael-type addition reaction of Ni(II) complex of dehydroalanine Schiff base **81** [82] and ethyl bromodifluoroacetate in the presence of copper for the synthesis of racemic 4,4-difluoroglutamic acid derivative were investigated. These reactions proceeded smoothly and gave the desired product **82** in 80% yield. By the disassembly of Ni(II) complex and *N*-protection, racemic Cbz-4,4-difluoro glutamic acid **83** was obtained in three steps from known compounds [83] (Scheme 30).

**Scheme 31:**
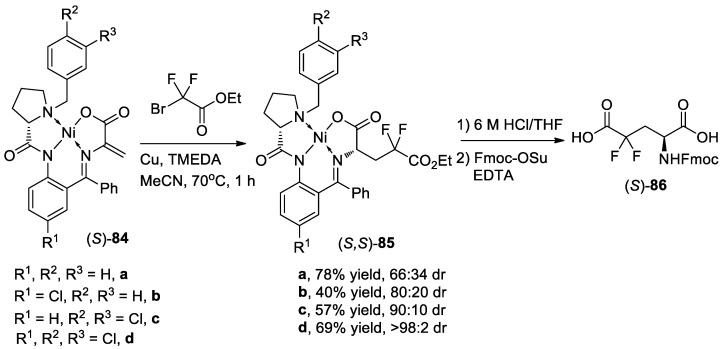
Asymmetric synthesis of difluoroglutamic acid derivative (*S*)-**86** via Michael-type addition reaction between chiral complex (*S*)-**84** and ethyl bromodifluoroacetate.

Based on the above results, four chiral Ni(II) complexes of dehydroalanine Schiff base were prepared and reacted with ethyl bromodifluoroacetate in the presence of copper to yield enantiomerically enriched Cbz-4,4-difluoro glutamic acid (*S*)-**83** [84]. As a result, it was found that the diastereoselectivity of this Michael-type addition reaction depends on the chlorine substitutions on the starting Ni(II) complexes and the Soloshonok ligand-derived trichloro-substituted Ni(II) complex of a dehydroalanine Schiff base (*S*)-**84d** afforded an attractive value of >98:2 diastereomeric ratio (Scheme 31).

**Scheme 32:**
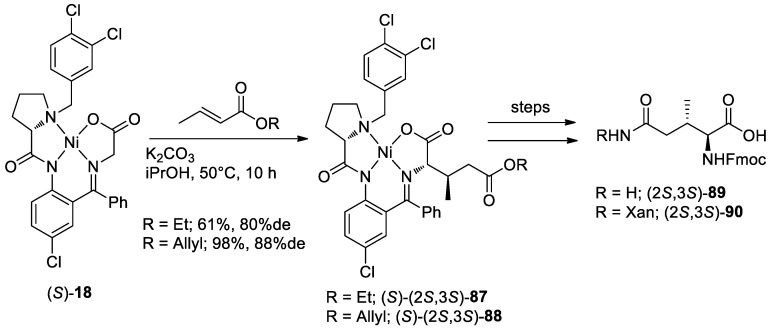
Asymmetric synthesis of optical active 3-Me-glutamine derivatives **89** and **90** via Michael-type addition reaction between chiral complex (*S*)-**18** and crotonate.

Practical new routes for the preparation of optical active 3-Me-glutamine derivatives were described using Michael addition reaction of Ni(II) complex of chiral Gly-Schiff base (*S*)-**18** (Scheme 32). These derivatives are classified as conformationally constrained tailor-made AAs and are found in several unique peptidyl natural products [85,86,87]. In particular, 3-Me-glutamine residue is a structural part of callipeltins O and Q isolated from a marine sponge [88]. Michael additions of (*S*)-Gly-Schiff base Ni(II) complexes of type **18** provide a reliable access to -substituted glutamic acid derivatives [46,47,52]. In the recent work, this approach was used for preparation of derivatives **89** and **90** needed for the total synthesis of some analogs of natural callipletins [88]. Complex (*S*)-(2*S*,3*S*)-**87** was prepared via Michael addition of (*S*)-Gly-Schiff base **18** and ethyl crotonate (61% yield and 80% de). After the disassembly of Ni(II) complex, followed by the Fmoc protection, target (*S*)-(2*S*,3*S*)-Fmoc-3-MeGlu-OH **89** was isolated in optically pure form. Likewise, (2*S*,3*S*)-Fmoc-3-MeGlu(Xan)-OH **90** was conveniently synthesized from allyl crotonate via (*S*)-(2*S*,3*S*)-**88**. In this case, several steps to change the protecting groups were needed to prepare the target AA suitable for solid-phase peptide synthesis [89].

## 7. Coupling Alkylation

**Scheme 33:**
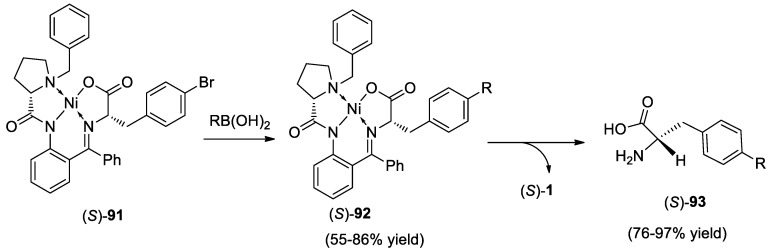
Suzuki reaction between (*S*)-**91** and different boric acids.

Tailor-made AA containing biphenyl groups are often used in the polymers, dyes and other compounds [3]. A variety of enantiomerically enriched non-protein (*S*)-α-amino acids with biphenyl groups in the side chain based on the Suzuki coupling reaction were obtained (Scheme 33) [90]. This Suzuki coupling reaction showed a wide substrate generality with regard to boronic acid derivatives. In most cases, the reactions furnished the products **92** in moderate to good yields (55–86%). Relatively high yields were recorded with phenyl- (86% yield) and biphenylboronic (83% yield) acids. The decomposition of complexes **92** and isolation of enantiomerically enriched target amino acids **93** were also carried out. It should be noted that some of amino acids could be crystallized directly from the acid hydrolysate by passing the stage of ion exchange purification (demineralization).

**Scheme 34:**
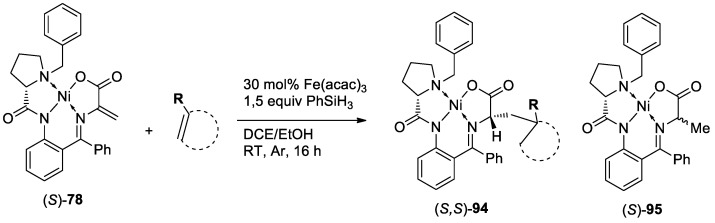
Fe-catalyzed coupling reaction of Ni complex **78**.

Tailor-made enantiopure α-AAs with γ-tertiary and quaternary carbon centers display various interesting biological activities [91,92]. Practical new routes for the preparation of these compounds via iron-catalyzed olefin-olefin coupling of dehydroalanine complex **78** were reported (Scheme 34) [93]. Stirring of Ni(II) complex **78** with 5 equiv of olefin in the presence of an inexpensive Fe(acac)_3_ mediator (30 mol%) and 1.5 equiv of phenylsilane in a mixed solvent of 1,2-dichloroethane (DCE)/EtOH at room temperature furnished product (*S*,*S*)-**94a** in a 90% yield (dr > 20:1). The formation of a by-product (*S*)-**95** was also observed with less than 5% yield. A variety of different olefins reacted very well with (*S*)-**78** affording products **94** in good yields (Scheme 34). The reaction also showed good diastereoselectivity, and in all cases the ratio of (*S*,*S*,X) to (*S*,*R*,X) was more than 15:1. Two gram-scale reactions with α-methyl styrene and methyl methyl(vinyl)phosphinate were also performed. The desired products (*S,S*)-**94** were isolated via standard silica chromatography in 93% (2.3 g) and 63% (1.6 g). Authors succeeded to isolate two enantiopure (*S*)-amino acids in 83% and 78% yields, respectively.

**Scheme 35:**
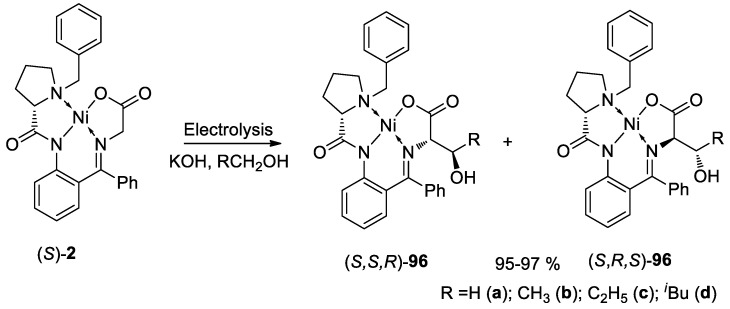
Electrochemical stereoselective oxyalkylation of the (*S*)-**2**.

A new electrochemical method for stereoselective hydroxyalkylation of (*S*)-**2** was elaborated by Levitskiy and co-authors (Scheme 35) [94]. Galvanostatic electrolysis of (*S*)-**2** was performed in a one-compartment electrochemical cell in alcohol solution in the presence of KOH, which acts as a deprotonating agent and a supporting electrolyte simultaneously. Electrochemical oxidation of the alkoxide anion leads to the corresponding carbonyl compound which reacts with the deprotonated (*S*)-**2** complex. It was found that the stereochemical outcome of the process is depended on the pH of the solution and the reaction time between the end of the electrolysis and the work-up procedure. Thermodynamically more stable (2*S*)-complex (for MeOH) and (2*R*,3*S*)-diastereomer (for the homologues) were formed at high pH with high diastereoselectivity (de up to 94%). In the media with low basicity, the (2*S*) complex (for MeOH) and (2*S*,3*R*)-diastereomer (for higher alcohols) can be obtained (de = 100%).

## 8. Dynamic Kinetic Resolution

Among various types of fluorinated tailor-made AAs, linear ω-CF_3_-AAs represent a family of structurally relatively simple, yet very useful compounds widely used in drug development and peptide research [95,96,97,98,99,100,101,102,103,104]. In particular, (*S*)-2-amino-5,5,5-trifluoropentanoic acid, a fluorinated analog naturally occurring in bacterial proteins norvaline [105], has been extensively used in the design of bioactive formulations [106,107,108,109,110,111,112] and protein research.

**Scheme 36:**
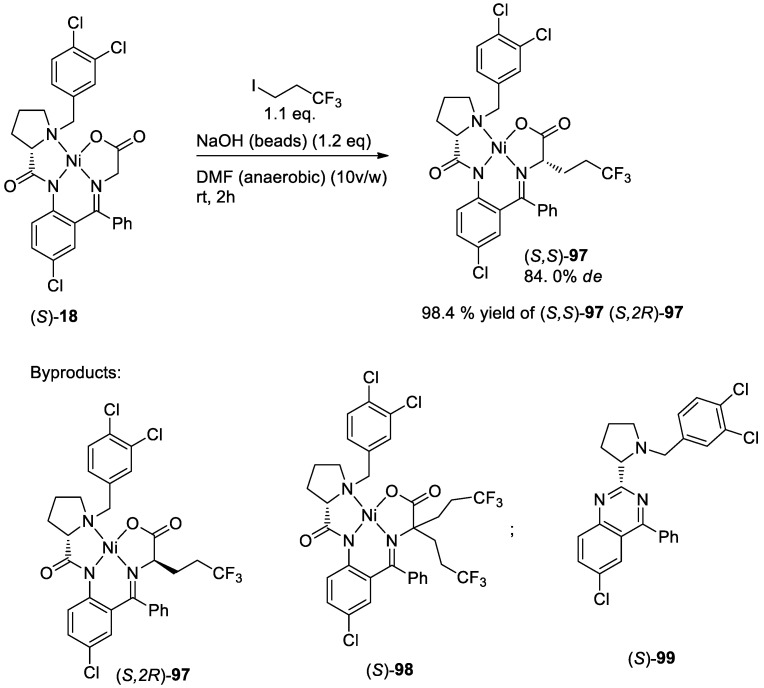
Alkylation of chiral glycine equivalent (*S*)-**18.**

In 2019, two alternative approaches were developed for the preparation of (*S*)-2-amino-5,5,5-trifluoropentanoic acid and its Fmoc-derivative [113]. The authors reported that the alkylation procedure could be successfully performed on a ~1 g scale, while the dynamic kinetic resolution was generally a more practical solution for scaled-up (~20 g) synthesis. Based on alkylation [16,36] and kinetic resolution [31,33] approaches, authors used trichloro-substituted ligands for the preparation of the target (*S*)-2-amino-5,5,5-trifluoropentanoic acid derivatives. In the case of alkylation of glycine complex (*S*)-**18,** 1,1,1-trifluoro-3-iodopropane was used as alkylation reagent. It should be mentioned that several byproducts including minor diastereomer (*S*)(2*R*)-**97**, bis-alkylated complex (*S*)-**98**, and 4-phenylquinazoline (*S*)-**99**, were also observed in these reactions. Application of a base in homogeneous form like NaOMe, solutions, or ground to powder NaOH led to noticeable decomposition of the alkylation reagent due to the favorable formation of CF_3_−CH=CH_2_ (Scheme 36).

Another way for the preparation of (*S*)-2-amino-5,5,5-trifluoropentanoic acid (**100**) was dynamic kinetic resolution (DKR) approach with tri-chlorosubstituted ligand (*S*)-**16** and racemic hydrochloric salt of the 2-amino-5,5,5-trifluoropentanoic acid (**100**) as the starting materials. The hydrochloric salt of the 2-amino-5,5,5-trifluoropentanoic acid was obtained from the commercially available *N*-acetyl derivative of **100** by acidic hydrolysis of the 6*N* HCl. In contrast to the alkylation procedure, DKR is usually conducted in an alcohol solvent and the process is not complicated by oxidative reactions leading to the byproducts of type (*S*)-**99**.

**Scheme 37:**
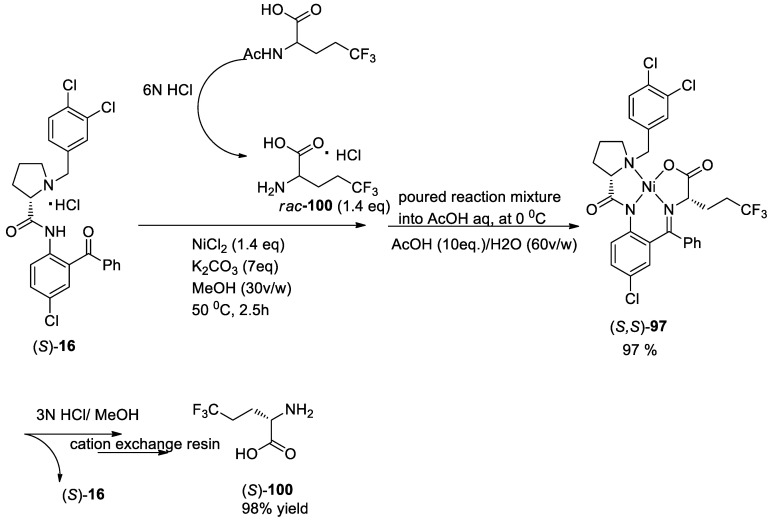
DKR of 2-amino-5,5,5-trifluoropentanoic acid (**100**).

The best yield of the product (*S,S*)-**97** (97.0%) was obtained with the application of 1.4 equiv excess of rac-**100**, NiCl_2_, and 7 equiv of the base. Most importantly, product (*S,S*)-**97** was obtained in a virtually diastereomerically pure form (99.5% d.e.). Moreover, authors examined the reproducibility of this method for small- as well as larger-scale reactions. For example, using 16.6 g of ligand (*S*)-**16**, they obtained 38.7 g of (*S,S*)-**97** (97% yield, 99.5% d.e.) (Scheme 37).

For the demonstration of the conventional preparative value of this method, authors performed a modified disassembly procedure and isolated the Fmoc-derivative of (*S*)-**100** amino acid with excellent chemical yield (93.3%) and enantiomeric purity (98.2% e.e.).

**Scheme 38:**
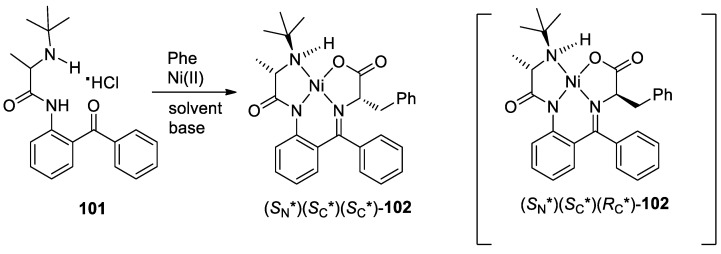
Reaction of **101** with phenylalanine.

Bremerich et al. reported an interesting way for the general asymmetric synthesis of α-amino acids using *N*-(2-benzoylphenyl)-2-(*tert*butylamino) propanamide (**101**) as chiral auxiliary by in situ formation of the corresponding Schiff bases with amino acids followed by complexation with Ni(II) ions and base-catalyzed thermodynamic equilibrium [114] (Scheme 38). Treatment of *N*-(2-benzoylphenyl)-2-(*tert*butylamino) propanamide (**101**) with racemic phenylalanine in the presence of Ni(II) ions afforded separable major product (*S*_N_*)(*S*_C_*)(*S*_C_*)-**102** and diastereomer (*S*_N_*)(*S*_C_*)(*R*_C_*)-**102**.

Under the optimal conditions, the reactivity and diastereomeric preferences of **101** complexations with various substituted phenylalanines were investigated. It was shown that the stereochemical outcome of the reactions of mono- and di-substituted phenylalanines was rather similar with >90% yields and diastereomeric ratios of about 95:5. Noteworthy, 3,5-di-trifluoromethyl-containing phenylalanine gave virtually complete diastereoselectivity. 3-hydroxy 4-nitro derivatives of phenylalanine were converted very well affording with excellent stereochemical control. To further expand this substrate generality, reactions of **101** with numerous α-amino acids bearing various side-chains were performed. Amino acids such as alanine, α-aminobutyric acid, norvaline, norleucine and leucine afforded diastereomeric products with both high diastereoselectivities (de ~95:5) and excellent chemical yields (88–99%).

**Scheme 39:**
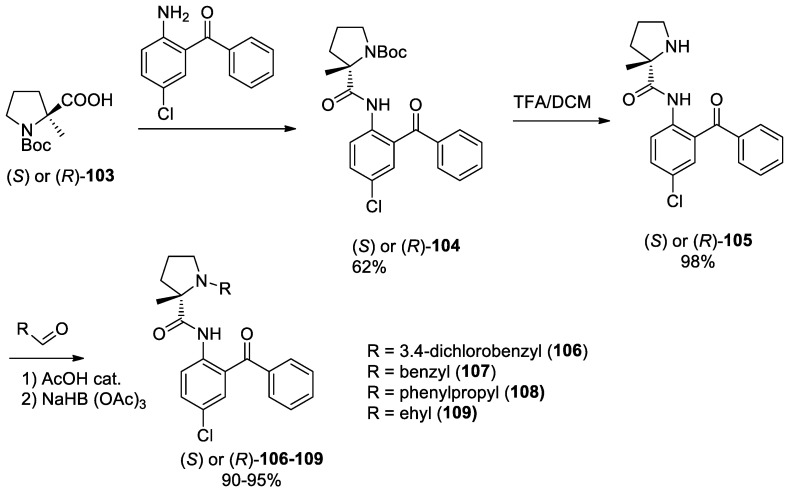
Synthetic approach to ligands (*S*)- and (*R*)-**106–109**.

Another way for the resolution of unprotected racemic AAs was developed by Zhou et al. with the use of a new α-methylproline-derived ligands [48]. They succeeded to modify the established synthetic procedure for the large-scale preparation of proline ligands (*S*)- and (*R*)-**106**–**109** includes the *N*-benzylation of free proline followed by a reaction with an *o*-aminobenzophenone derivative [59,82]. They implemented a procedure based on *N*-Boc-α-methylproline (**103**). Using this approach, it was found this reaction quite reliable, reproducible, and operationally convenient for scaling up (Scheme 39).

**Scheme 40:**
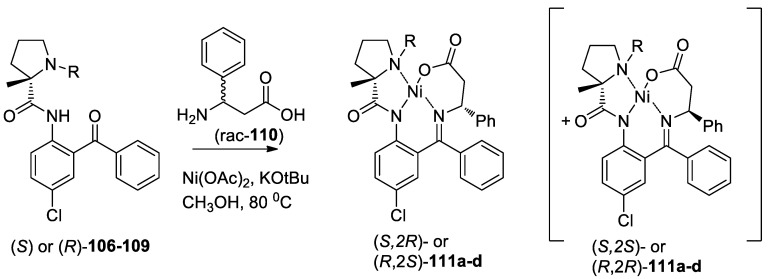
Chemical resolution of β-phenyl-β-alanine **110** with new chiral ligands **106**–**109**.

Ligands **106**–**109** were then tested in the chemical resolution of β-phenyl-β-alanine (Scheme 40). Based on the results, authors concluded that the nature of the substituent on the proline nitrogen atom does not show an obvious effect on the stereochemical outcome. The nickel complexes **111** were isolated in 54–83% yields and 95:5 to 88:12 diastereoselectivities. The observed subtle differences can be attributed to the steric effects of the substituent on the stabilities of the major and minor products under strongly basic conditions.

Various substituted β-amino acids were tested. In the cases of β-phenyl-β-substituted racemates, the corresponding diastereomers were obtained with average chemical yields of ca. 90% and good diastereomeric preferences of more than 90:10. The presence of halogen atoms, alkyl, methoxy or trifluoromethyl group did not show any noticeable influence on the stereochemical outcomes. Racemic β-amino acids containing heterocyclic moieties, such as pyridyl, thienyl or naphthyl groups, reacted with ligand **109** without any complications to furnish products **111** with excellent yields and diastereomeric ratios (chemical yield 80–95%, d.r. ~98:2). In the aliphatic series, racemates bearing straight chains such as methyl, ethyl and *n-*butyl groups reacted well with ligand **109** to afford diastereomers with excellent stereochemical outcomes (chemical yield 76–95%, d.r. ~95:5). Furthermore, authors successfully applied the method to obtain the essential intermediate β-phenyl-β-alanine (92% chemical yield, and >99% e.e.) in the synthesis of the anti-HIV drug maraviroc in an overall yield of 49%, which possibly makes it of great interest to industry.

## 9. Miscellaneous Reactions

**Scheme 41:**
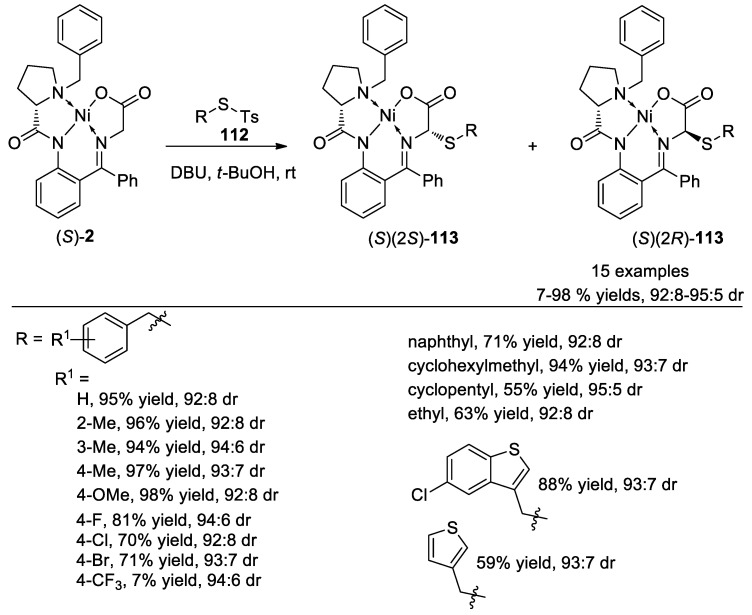
Asymmetric synthesis of chiral α-benzyl mercaptoglycine derivatives.

Due to the special role of α-substituted mercaptoglycine derivatives in many bioactive compounds [115,116], the development of efficient methodologies for the synthesis of α-substituted mercaptoglycine derivatives has attracted great attention. In 2017, Wang and co-authors developed a synthetic method for the preparation of the chiral α-substituted mercaptoglycine derivatives (*S*)(2*S*)-**113** via an asymmetric substitution reaction of Ni(II) complex (*S*)-**2** with *S*-substituted 4-methylbenzenesulfonothioates as the sulfur sources (Scheme 41) [117]. After careful optimization of reaction conditions, they found that the reaction could proceed very well with 2.0 equiv of DBU as a base in *t*-BuOH at room temperature. The reaction showed good functional group tolerance, and total fifteen examples of *S*-substituted 4-methyl-benzenesulfonothioates were examined with good chemical yields (7–98%) and excellent diastereoselectivities (92:8–95:5 d.r.). However, the substrate bearing a 4-trifluoromethylbenzyl strong electron-withdrawing group, was found to be unsuitable for this reaction and less than 10% yield was found. It should be mentioned that the substrates featuring cycloalkyl and ethyl groups were also well tolerated in this reaction.

**Scheme 42:**
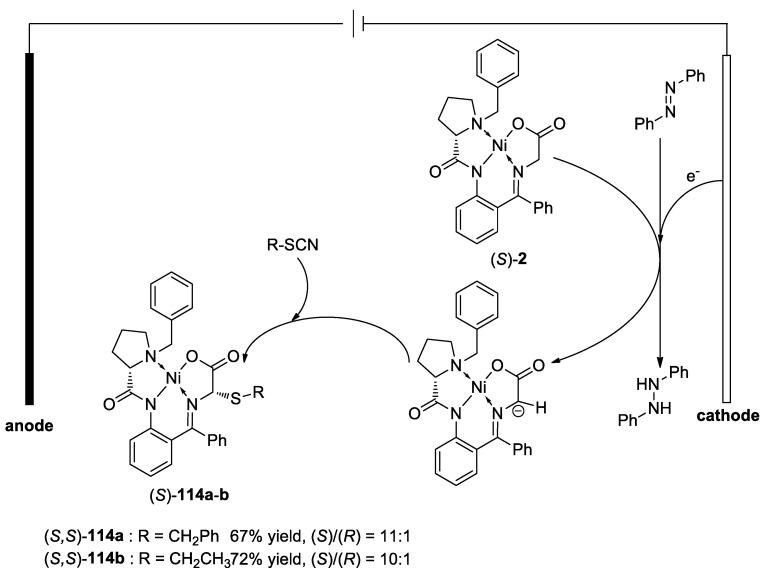
Electrochemical reaction of Ni complex and thiocyanates.

In 2017, Magdesieva and co-workers developed a one-pot method for the asymmetric synthesis of α-thioalkylated amino acids under electrochemical reaction conditions (Scheme 42) [118]. 

This thioakylation reaction of Ni complex (*S*)-**2** was conducted in a divided *H*-type cell in the presence of azobenzene at a constant potential (−1.40 V vs. Ag/AgCl/KCl), which proceeded smoothly to give the corresponding thioalkylated amino acid derivatives in up to 72% yield and 11:1 diastereoselectivity. Two alkyl thiocyanates, benzyl and ethyl thiocyanate were employed in this reaction, and the same level of yield and diastereoselectivity were found. The absolute configuration of the newly generated carbon center is (*S*), which has been confirmed by the x-ray single crystal analysis of complex **114b**.

According to the results from cyclic voltammetry (CV) experiments, one possible mechanism was proposed, which proceeds via the sequence of reduction of azobenzene at cathode to give the radical anion, deprotonation of Ni complex and substitution of thiocyanate. It was worth mentioning that thiocyanates acted very well as the thioakylation reagents in this electrochemical reaction, and could avoid the epimerization of the product when common bases were used.

**Scheme 43:**
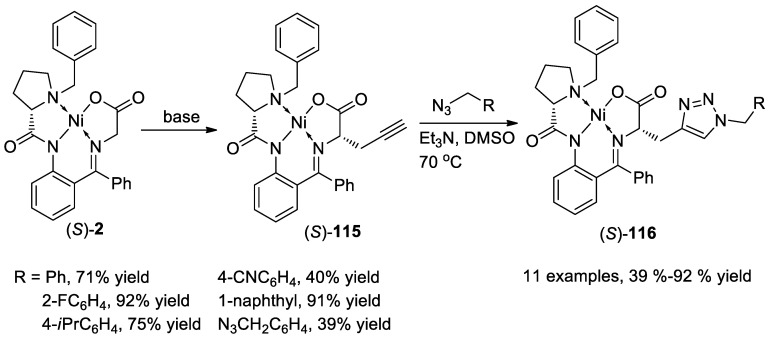
Synthesis of triazoles via click reaction of Ni complex (*S*)-**115.**

In 2018, Belokon and co-workers [119] reported the simple approach for the synthesis of amino acids bearing a 1,2,3-triazole functionality via a copper(I) iodide catalyzed click reaction between alkynyl Ni(II) complex (*S*)-**115** and alkyl azide (Scheme 43). It should be mentioned that the Ni(II) complex (*S*)-**115** could be obtained easily via the alkylation reaction of propargyl bromide with Gly-based Ni complex (*S*)-**2** in the presence of base. The cycloaddition proceeded smoothly with triethylamine as a base in DMSO at 70 °C for 7 h, affording the chiral triazole products **115** in 39–92% yields. The reaction showed good functional group tolerance. Notably, diazide also could react with complex (*S*)-**116**, resulting in the bis-triazole Ni(II) complex in 39% yield.

**Scheme 44:**
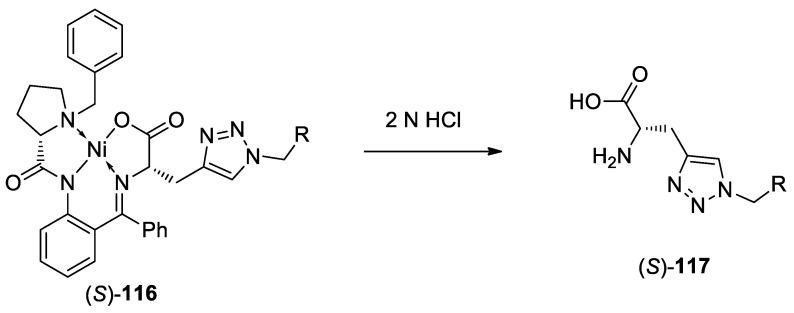
Disassembly of the Ni complex (*S*)-**116** to give free amino acid (*S*)-**117.**

The disassembly of (*S*)-**116** to free amino acid (*S*)-117 was developed in this work. The desired triazole containing amino acids (*S*)-117 could be isolated in excellent yields and high enantioselectivities (>99% ee) under the acidic conditions (Scheme 44). The chiral ligand was easily separated and could be used for the next synthesis cycle.

**Scheme 45:**
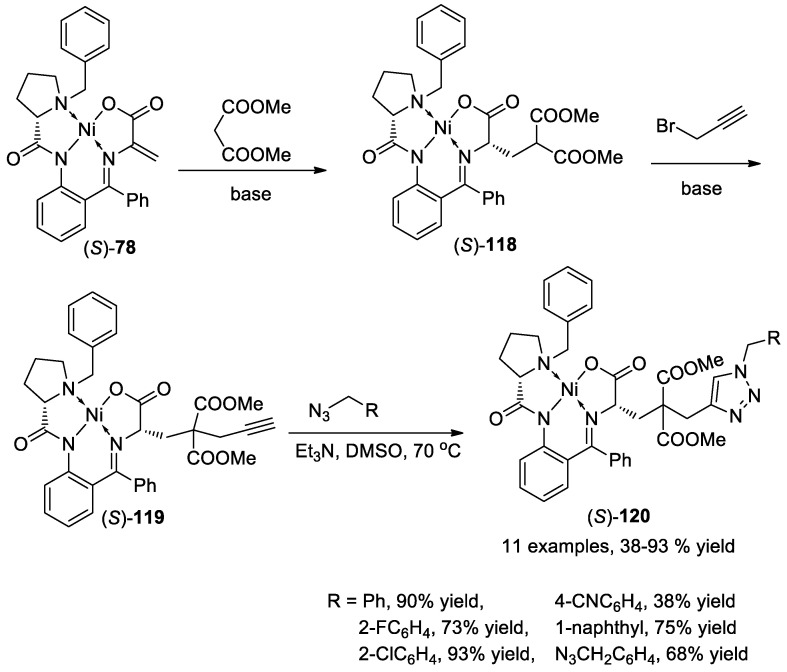
Synthesis of Ni(II) complex (*S*)-**119** and its click reaction.

The authors extended this click reaction to another Gly-based Ni complex (*S*)-**119** under the same reaction conditions. The Ni(II) complex (*S*)-**119** was prepared via a two-step synthetic method. The Ni(II) complex (*S*)-**118** was prepared by the Michael addition of malonic ester to dehydroalanine (*S*)-**78** [120]. Then, the alkylation of (*S*)-**118** with propargyl bromide in the presence of base afforded the Ni(II) complex (*S*)-**119** in 95% yield and d.r. > 99:1 (Scheme 45). The click reaction of (*S*)-**119** with eleven examples of azides was successfully achieved in the presence of triethylamine with CuI as a catalyst at 70 °C, affording the chiral triazole products in 38–93% yields. For most cases of the azides, good yields were observed. However, for a strong electron withdrawing-group on the azide-bearing phenyl, such as 4-cyano, a poor yield was found (40%).

**Scheme 46:**
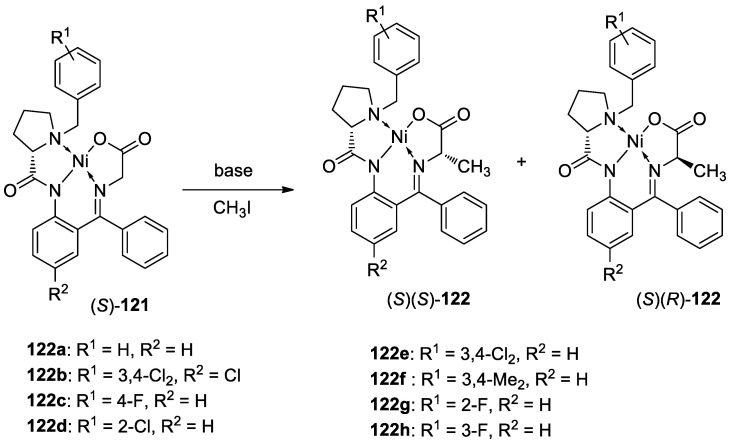
Structure of Ni complex **121a-h** and their methylation derivatives.

In 2017, Soloshonok, Liu [121] and co-workers reported their studies on the effect of substituents on glycine-based Ni complexes on stereochemical preferences. A series of glycine-based Ni complexes (**121a**–**h**) containing different substitutions on the proline *N*-benzyl group or *o*-amino-benzophenone moiety were synthesized. Also, the methylation of complexes **121a**–**h** with the use of MeI in the presence of base was conducted and the corresponding Ni complexes (**122a**–**h**) were obtained (Scheme 46). They assumed that the substitutions on the proline *N*-benzyl and o-amino-benzophenone moieties are far away from the carbon center of glycine moiety and most probably have no obvious electronic and steric effects on the stereochemical outcome of the related asymmetric reactions. However, the existence of these substitutions may lead to changes of the intramolecular interactions and Ni(II) chelating models. Consequently, thirteen single crystals of Ni(II) complexes were prepared and subjected to the x-ray crystallographic analysis. The analysis of crystal structures clearly shows that almost no aromatic-aromatic interactions were detected for complex **121a**, while obvious interactions were found for the substituted complex **121b**. Analysis of complexes (*S*)(2*S*)-**122a** and **122b** found that the distance between the aryl C-H hydrogen atom and the *o*-carbon of the benzophenone ring in **122b** is greater than in unsubstituted complex **122a**, which indicates **122b** is more thermodynamically stable. The same phenomena are found in those substituted complexes **122b**–**d**. These findings could be used for the design of new Ni complex for the asymmetric synthesis of amino acids.

**Scheme 47:**
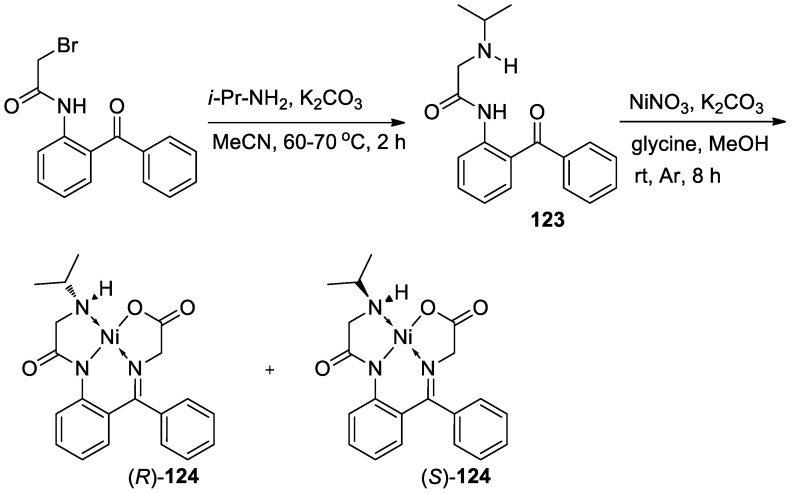
Synthesis of ligand **123** and racemic glycine Schiff base Ni(II) complex **124**.

Han [122], Soloshonok, and co-workers designed a new glycine-based Ni(II) complex, which contains only one single-nitrogen stereogenic center. This new Ni complex was synthesized starting from *N*-(2-benzoylphenyl)-2-bromoacetamide and isopropylamine (Scheme 47). The obtained amine intermediate **123** was converted into the designed Ni complex **124** via a reaction with nickel nitrate hexahydrate and glycine in the presence of K_2_CO_3_. The new Ni complex was used for measuring the Ni(II) complex configurational stability via high-performance liquid chromatography (HPLC)-assisted evaluation. The results showed that the stability mainly depends on the solvent, which ranges from t_½_ less than 5 min to completely stable (t_½_ 90 year) at room temperature.

**Scheme 48:**
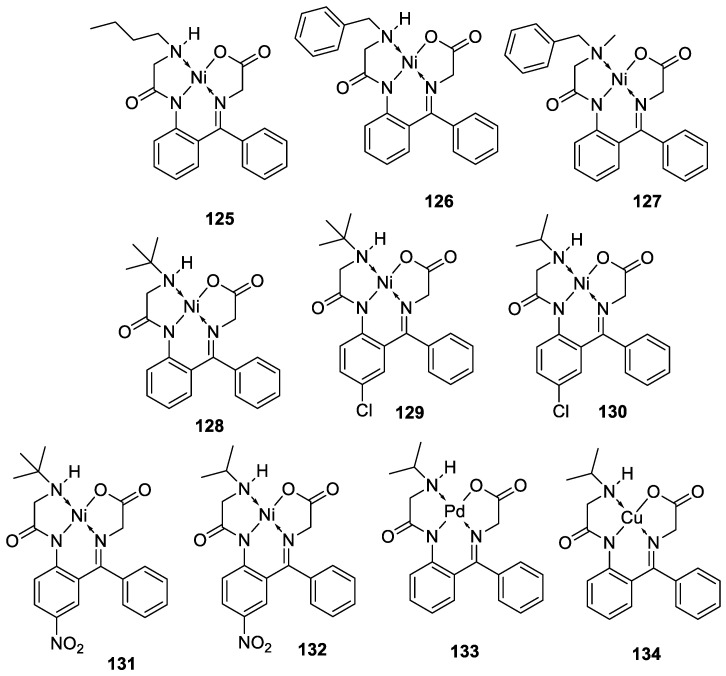
Structures of complex **125–134**.

Shortly after that work, the same authors extended their work to other metal(II) complexes of α-amino acid Schiff bases to investigate the general rule on the effect of substituents on the configurational stability of the stereogenic nitrogen in such type of metal(II) complexes (Scheme 48) [123]. They synthesized ten complexes with variations of amino group, substitution on *o*-amino-benzophenone and transition-metal by using of the similar method reported previously. Generally, Cu(II) complex is obviously less stable as compared with Ni(II) and Pd(II); the *N*-methylamine-based complex (**127**) is more stable than those with N-H type complexes (**125**,**126**).

## 10. Conclusions

The results discussed in this review article clearly underscore the leading position of Ni(II) complexes as the methodology of choice for asymmetric syntheses of tailor-made AA. The variety of chiral tridentate ligands and reaction chemistry continue to grow. While our aim was to present as comprehensive treatment of the subject as possible, we eventually missed some of the relevant data that were kindly pointed to us during the profound and very thoughtful refereeing of this work. These include Michael additions of chiral glycine Schiff base Ni(II)-complex with 1-(1-phenylsulfonyl)benzene [124], synthesis of β-substituted-tryptophans via three-component reactions of glycine Ni(II)-complex [125], synthesis of β-substituted α,γ-diaminobutyric acid derivatives via Michal addition reactions [126] and an approach to γ-amino acids via Ni-catalyzed carbodifunctionalization of *N*-vinylamides [127]. Besides, novel application of amino acids in functional supramolecular materials [128,129] might also be of interest. The readers should consult these references for a more comprehensive overview of the developments in the field. Several notable trends can be pointed out as they, most likely, will define future research in this area. First of all, the readers can see the application of Ni(II) complexes as protective platform for the modification of side chain of the already assembles AAs. This trend is exemplified by the Suzuki coupling reactions and click chemistry. Compared to conventional types of protection, the assembly/disassembly of the corresponding Ni(II) complexes present a more simple and general solution. One may expect this line of application of Ni(II) complexes will be used by many research groups. Another interesting trend is a growing interest in electrochemical reactions. This is, probably, a reflection of “green” chemistry mentality deservingly taking roots in chemical practice. Also, we would like to mention the interest in more fundamental aspects of Ni(II) complexes structure and stereogenic properties, in particular related to the stereogenic nitrogen, its configurational stability and overall effect on the stereochemical outcome of the homologation reactions. Finally, most important in our opinion trend, is application of Ni(II) complexes methodology on large, near industrial scale. Some reactions have been conducted on multi-kilogram scale underscoring true practicality of this approach. One may confidently conclude, the discussed in this article developments over the last four years, bode well for future growth and methodological dominance of Ni(II) complex chemistry for practical preparation of structurally diverse tailor-made AAs for exciting applications in medicinal chemistry and drug design.
